# Evolution of the Thermopsin Peptidase Family (A5)

**DOI:** 10.1371/journal.pone.0078998

**Published:** 2013-11-27

**Authors:** Neil D. Rawlings

**Affiliations:** 1 Wellcome Trust Sanger Institute, Hinxton, Cambridgeshire, United Kingdom; 2 European Molecular Biology Laboratory, European Bioinformatics Institute, Hinxton, Cambridgeshire, United Kingdom; Stanford University, United States of America

## Abstract

Thermopsin is a peptidase from *Sulfolobus acidocaldarius* that is active at low pH and high temperature. From reversible inhibition with pepstatin, thermopsin is thought to be an aspartic peptidase. It is a member of the only family of peptidases to be restricted entirely to the archaea, namely peptidase family A5. Evolution within this family has been mapped, using a taxonomic tree based on the known classification of archaea. Homologues are found only in archaeans that are both hyperthermophiles and acidophiles, and this implies lateral transfer of genes between archaea, because species with homologues are not necessarily closely related. Despite the remarkable stability and activity in extreme conditions, no tertiary structure has been solved for any member of the family, and the catalytic mechanism is unknown. Putative catalytic residues have been predicted here by examination of aligned sequences.

## Introduction

Understanding the progression of evolution within a protein family can be very useful for understanding the functions of individual proteins. It is particularly useful to identify orthologues where function is often conserved. However understanding the evolution of a family can be made difficult to interpret because lateral gene transfer may occur between distant parts of the evolutionary tree. A lot of work has been carried out to understand the function and evolution of peptidases in the MEROPS database [1]. Whilst developing the software to help identify unexpected and unusual presences of peptidases within genomes, peptidase family A5 (the thermopsin family) was regularly used as an example, because it is a family with an ideal number of homologues for manually confirming the results that the software generated. The discovery of lateral gene transfers in the family, described here, was an unexpected result and prompted further study. Horizontal gene transfers were thought not to occur in archaeae until the genome of *Methanosarcina mazei* was completely sequenced and considerable genetic transfer between bacteria and the archaean was detected [2]. The recent discovery of plasmids in *Thermococcus* strains and their similarity to *Pyrococcus* viruses has provided a mechanism for lateral gene transfer in archaea [3].

Thermopsin is a little known peptidase isolated and characterized from *Sulfolobus acidocaldarius* [4], [5]. *S. acidocaldarius* is a thermophilic archaean originally isolated from the hot springs at Yellowstone National Park, USA and thrives at pH 2 and 70°C, and consequently thermopsin activity is optimal at pH 2 and 50–65°C, though proteolytic activity is observed over a much wider pH range (0–12). Activity drops off above 65°C and the enzyme is working at reduced activity at the optimal temperature for growth of the archaean.

Thermopsin is thought to be an aspartic peptidase, mainly because it is inhibited by inhibitors of aspartic peptidases such as pepstatin, diazoacetylnorleucine methyl ester and 1,2-epoxy-3-(*p*-nitrophenoxy)propane. Pepstatin is a competitive inhibitor, with a *K*_i_ of 10^−7^ M, which compares favourably with the inhibition of the pepsin homologue cathepsin D (*K*_i_ of 10^−10^ M) [6], except that for members of the pepsin family, inhibition is non-competitive. The active site residues of thermopsin are unknown. Thermopsin is synthesized as a precursor, containing a signal peptide and a short propeptide of approximately eleven residues. Activation is probably by self-cleavage of the Leu41+Phe bond. There is a single cysteine, but activity appears not to be thiol-dependent, because sulphydryl-modifying reagents do not act as inhibitors. There are eleven potential glycosylation sites, and the observed molecular mass (46,000–51,000) is much greater than the calculated mass (32,651). These data imply that thermopsin is a secreted protein that is heavily glycosylated, and that its function is to degrade proteins in the extracellular milieu for the provision of nutrients [7].

No tertiary structure for any member of the thermopsin family has ever been solved and the molecular fold is unknown.

There are a number of homologues of thermopsin present in a wide variety of archaeans, and what makes this family of peptidases (known as A5 in the *MEROPS* classification system [8]) interesting is that it is the only family of peptidases restricted to the Archaea. In this paper, I will discuss evolution within family A5 and how this compares to the taxonomy of the Archaea.

## Materials and Methods

### Detection of Homologous Sequences

The protein sequence of the peptidase domain, residues 32–330, of thermopsin from *Sulfolobus acidocaldarius* was submitted to the National Center for Biotechnology Information (NCBI) BlastP server (http://blast.ncbi.nlm.nih.gov/Blast.cgi) and homologues in the non-redundant protein sequence database were detected. A match was considered statistically significant if the E-value was 0.001 or less. In addition, the genomic Blast at NCBI (http://www.ncbi.nlm.nih.gov/sutils/genom_table.cgi) was also used to search specific archaean proteomes where a homologue was suspected but apparently absent.

### Secondary and Tertiary Structure Prediction

The JPred server (http://www.compbio.dundee.ac.uk/www-jpred) was used to predict secondary structure. The phyre (Protein Homology/analogY Recognition Engine) server (http://www.sbg.bio.ic.ac.uk/~phyre/) was used to predict possible relationships to existing tertiary structures [9]. The Muster website (http://zhanglab.ccmb.med.umich.edu/MUSTER) was used to model structures by threading the thermopsin sequence onto existing PDB structures [10].

### Determination of Evolutionary Pathway

The evolutionary pathway for the family was calculated from the taxonomic classification of the all the organisms with homologues, on the assumption that the existing taxonomy reflects the evolution of the organisms. Unclassified archaeans were excluded from the analysis. Once the family had been assembled a Perl script was written to retrieve the NCBI Taxonomy classification for each organism and for all the archaean species with completely sequenced genomes. The program reads from genus to phylum, comparing the child taxa immediately below and calculating the number of genes likely to be derived from the parent taxon. To be maximally parsimonious, a number is calculated for the parent taxon only if more than half of the child taxa possess a homologue. The program reports: the highest taxon possessing homologues, which should correspond to where the family originated; unexpected presence of homologues, which may be derived from horizontal gene transfers; gene losses and gene gains.

### Taxonomic Tree

The taxonomic tree was generated on the Interactive Tree of Life (IToL) website (http://itol.embl.de/). The tree was generated from a file containing the NCBI taxonomy (http://www.ncbi.nlm.nih.gov/taxonomy) identifiers for all archaeans for which the genome had been completely sequenced (at the time writing) plus any archaeans with a thermopsin homologue. Optimal growth conditions were extracted from the NCBI Genomes database (http://www.ncbi.nlm.nih.gov/genome/) or MicrobeWiki (http://microbewiki.kenyon.edu/).

### Phylogenetic Tree

An alignment of the amino acid sequences of thermopsin homologue peptidase domains (*i.e.* without the signal and activation peptides) was made using ClustalW [11]. The alignment was submitted to FastTree [12] to generate a neighbour-joining tree with 100 bootstraps. The tree was plotted as a circular cladogram using IToL.

## Results and Discussion

### Homologues Detected

Searches of the NCBI non-redundant protein sequence database and proteomes from archaea revealed 79 homologues from 21 different species. The species with most homologues is *Sulfolobus islandicus* with seven. Because the active site residues of thermopsin are unknown, it is not possible to distinguish active peptidases from non-peptidase homologues. A pHMMER search of the UniProt database using the HMM server (http://hmmer.janelia.org) and submitting the alignment of protein sequences of the homologues returned by the blastp search, failed to find any more significant matches.

### Potential Active Site Residues

On the assumption that thermopsin is an aspartic peptidase, and given that it does not dimerize to form an active peptidase, then at least two aspartic acid residues should be conserved. In the alignment of 75 homologues (Fig. S1), the following aspartic acid residues are well, but not absolutely, conserved: Asp129 (in the motif QDV), conserved in 28 sequences; Asp144 (in the motif DNVWN), conserved in 48 sequences; Asp228 (in the motif YDKITI), conserved in 61 sequences; Asp257 (in the motif DAELV), conserved in 70 sequences; Asp302 (in the motif Asp-Thr-Gly/Glu/Ala), conserved in 61 sequences. Asp129 is replaced by Asn in 43 sequences, and as it has been postulated that deamidation of Gln to Glu in some glutamic peptidases may occur [13] a similar process may happen to convert Asn to Asp. It is not unusual for a family of peptidases to contain non-peptidase homologues in which active site residues have been replaced, so 100% conservation is not to be expected.

There is also the possibility that thermopsin is not an aspartic peptidase but an enzyme of a different catalytic type. The other residues that are well conserved and which might be part of an active site are Gln109, Asn111, Gln128, Asn148 and Glu305. In glutamic peptidases, there is a catalytic dyad composed of a Gln and a Glu; however, thermopsin is unlikely to be a glutamic peptidase because glutamic peptidases such as scytalidopepsin B are insensitive to pepstatin [14]. There is no well conserved His, Ser or Cys residue; in fact *Sulfolobus* thermopsin has no histidine residues in the mature peptidase. Thr150 is conserved in 51 sequences and replaced with Ser in 22 sequences: it is known that in some N-terminal nucleophile hydrolases that the nucleophilic threonine can be replaced by serine, both naturally and artificially, and the peptidase is still active [15], [16], however, there is no evidence of processing to generate an N-terminal residue at this position.

### Structure Prediction

Thermopsin is predicted to be mainly an all-beta protein, which would be consistent with a pepsin-like fold [17], with short helices predicted in the signal peptide, at residues 125–131, 253–261 and 275–281 (by at least two of the three prediction methods used by JPred). Of the five aspartic acids that might be active site residues, Asp129 and Asp257 are predicted to be within helices, and Asp144 and Asp228 within strands. Asp302 is predicted to be within random coil. If thermopsin adopts a pepsin-like fold, then the active site residues would be expected to be within strands.

Pepsin is derived from a gene duplication/fusion event that is so ancient it is only evident from the tertiary structure. The only evidence of this duplication in the protein sequence is the conservation of the Asp-(Thr/Ser)-Gly motifs that include the two active site aspartic acid residues. If thermopsin adopts a pepsin-like fold, then the two-fold symmetry should also be apparent in the structure and in the motifs around the active site residues. Only the motif around Asp302 (Asp-Thr-Gly/Glu/Ala) is similar to the motif in pepsin.

Other aspartic peptidases with known folds that are different to pepsin are the preflagellin peptidase from *Methanococcus maripaludis*, the gpr endopeptidase from *Bacillus megaterium*, and omptin from *Escherichia coli*. The preflagellin peptidase is a transmembrane protein consisting mainly of helices [18]. Omptin is also a transmembrane protein but consists of a ten-stranded anti-parallel beta-barrel structure [19]. The structure of the gpr endopeptidase precursor [20] shows a homotetramer with the monomers arranged such that there is a 9 Å channel in the middle. The structure of the monomer is of seven-stranded beta sheet surrounded by helices. Thermopsin is not associated with membranes and is therefore unlikely to adopt the fold of either preflagellin peptidase or omptin, and if it is truly an all-beta protein and monomeric is unlikely to have a fold similar to that of the gpr endopeptidase.

The Phyre website failed to find any structural fold that was significantly related to the thermopsin sequence. The best match was to the pectate transeliminase family with an E value of 3.1. The MUSTER website was able to thread the thermopsin sequence onto structures with a beta-propeller-like fold, but the Z scores were very low and not statistically significant. The conserved aspartic acid residues were not placed in regions that looked like active sites. These results suggest that the fold of thermopsin is unlike that of any existing aspartic peptidase, and likely to be novel.

### Other Putative Peptidase Families Restricted to Archaea

Although family A5 is the only peptidase family known to be restricted to archaea, there have been claims for two other peptidases found only in archaea. A protein from *Sulfolobus solfataricus* has been claimed to be an intracellular peptidase, with a preference for cleavage after glutamic acid. Because it is sensitive to thiol-blocking reagents it has been claimed to be a cysteine peptidase with distant homology to caspases, but the possibility that it is a thiol-dependent metallo- or serine peptidase has not been examined [21]. From the N-terminal sequence, a protein of only 95 residues was identified in the proteome, which would make this the smallest peptidase known. Cys18 was predicted to be the nucleophilic residue, and given that the active site residues in a caspase are in the order His, Cys, a subdomain bearing the active site His seems to be missing. There are many homologues amongst archaea, and the putative catalytic Cys is not conserved.

Putative protein PAE0478 from *Pyrobaculum aerophilum* genomic sequence [22] has been claimed to be a metallopeptidase because of the presence of the Met-zincin-like motif HEFGHNLGLRH (see UniProt: Q8ZZ23). However, no activity has ever been biochemically shown, and there are no other homologues.

### Evolution of Family A5

**Figure 1:**
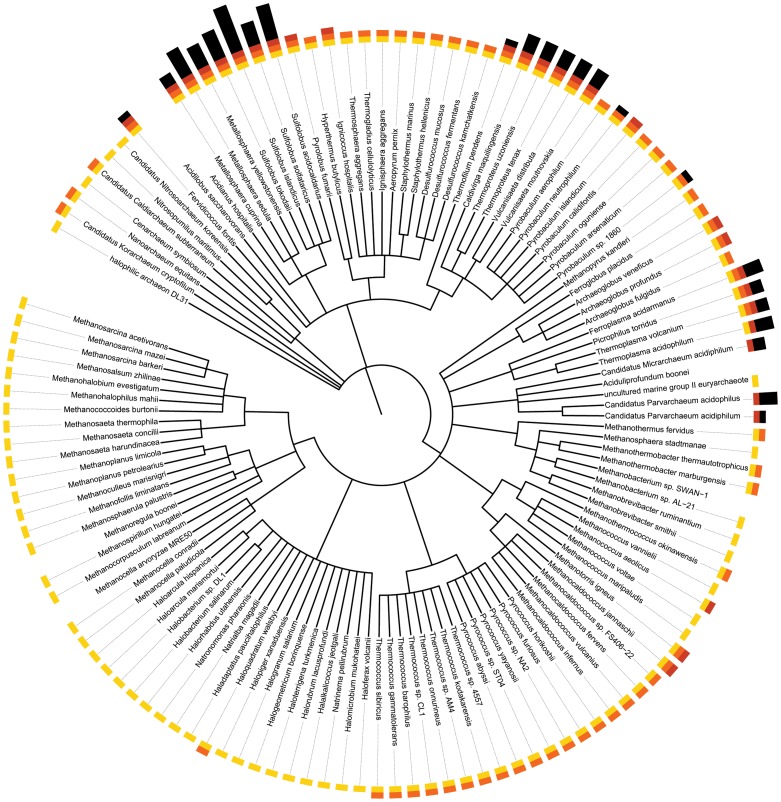
Distribution of thermopsin homologues amongst archaea with completely sequenced genomes. The phylogenetic tree is based on the classification from the NCBI Taxonomy database. Tips are annotated with coloured bricks as follows: yellow, completely sequenced genome; orange, hyperthermophilic; red, acidiophilic; black, presence of a thermopsin homologue. The number of black bricks indicates the number of thermopsin homologues.

As can be seen from Fig. 1, a thermopsin homologue is only found in an archaean that is an acidophile, and usually a hyperthermophile as well. The only species that are not hyperthermophiles and yet have a thermopsin homologue are *Thermoplasma acidophilum*, *Candidatus Micrarchaeum acidiphilum*, *Candidatus Parvarchaeum acidophilum* and *Candidatus Parvarchaeum acidophilum*, and for the uncultured archaeans optimum pH for growth has not been determined. The reverse, however, is not true: there are acidophiles that are also hyperthermophiles but which do not possess a thermopsin homologue, including *Pyrolobus fumarii*, Ignicoccus hospitalis, *Pyrobaculum islandicum*, *Archaeoglobus veneficus, Archaeoglobus profundus, Methanotorris igneus* and *Methanocaldococcus jannaschii*.

From comparing the distribution of the thermopsin family with the taxonomic classification of the Archaea (see Fig. 1), the calculated origin of the thermopsin family is the class Thermoplasmata in the phylum Euryarchaeota. This is the highest clade where species with a homologue exceed those without. In fact, all four completely sequence genomes within this class have homologues. This is a surprise, because thermopsin has been characterized from *Sulfolobus acidocaldarius*, which is a member of the order Sulfolobales, the class Thermoprotei and the phylum Crenarchaeota. Presence in the order Sulfolobales is likely to be a result of an ancient horizontal gene transfer between an ancestral Euryarchaeote and an ancestral Crenarchaeote. There has also been a substantial increase in genes in the order Sulfolobales, with three rounds of gene duplications to give four homologues in the ancestor of the order.

Unexpected presences include within the family Thermofilaceae, the genera *Caldivirga*, *Thermoproteus* and the species *Pyrobaculum* sp. 1860. In the genus *Caldivirga*, there has also been a lineage specific expansion to three genes. The genus *Thermoproteus* has also undergone an expansion, with two homologues.

Although no losses have resulted in the absence of a homologue, there are several instances where the number of paralogues has been apparently reduced. Species in the family Picrophilaceae have fewer homologues than is predicted for an ancestor in the order Thermoplasmatales (one homologue compared to three). Members of the genus *Metallosphaera* have fewer homologues than is predicted for an ancestor of the family Sulfolobaceae (two homologues compared to four). Gene losses are also predicted for several species: *Sulfolobus islandicus* and *S. solfataricus* (one homologue when the ancestor in the genus is predicted to have five); *Thermoproteus neutrophilus* (one homologue compared to two for the ancestor of the genus); and *Thermoplasma volcanium* (one homologue when the ancestor of the genus is predicted to have three).

Gains because of gene duplications have occurred in the genera *Acidianus* and *Sulfolobus*, which have six and five homologues compared to four predicted for the family Sulfolobaceae; the species *Metallosphaera sedula* has five homologues compared to the two predicted for the genus; the species *Sulfolobus acidocaldarius*, which has six homologues compared to the five predicted for the genus; and the species *Thermoproteus tenax* and *T.uzoniensis,* both with three homologues compared to one predicted for the genus.

Thermopsin homologues derived from unclassified archaeans were not included in this tree. These include sequences from the uncultured *Micrarchaeum acidophilum*, *Parvarchaeum acidophilus*, and the nanoarchaeote Nst1.

Nanoarchaeote Nst1 is an organism collected from a thermal pool at Yellowstone National Park [23], which is also the source of the only other known nanoarcheote, *Nanoarchaeum equitans. N. equitans* has a much reduced genome and lacks a thermopsin homologue. Without further data it is impossible to tell if the protein from nanoarchaeote Nst1 has arisen because of a horizontal gene transfer, or if *N. equitans* has lost the gene for a thermopsin homologue that was present in an ancestor of the phylum Nanoarchaeota. It could be argued that if the ancestral nanoarchaeote possessed a thermopsin homologue, then this would dramatically alter the predicted evolutionary history, because three of the five phyla would possess a homologue, suggesting that the origin of the thermopsin gene was the ancestral archaean, and the gene was lost from the phyla Korarchaeota and Thaumarchaeota. However, the fact that only species from one class in the Euryarchaeota possesses a homologue (Thermoplasmata) and that species from the other eight classes do not, implies that the ancestral euryarchaeote did not have a thermopsin homologue.

**Figure 2:**
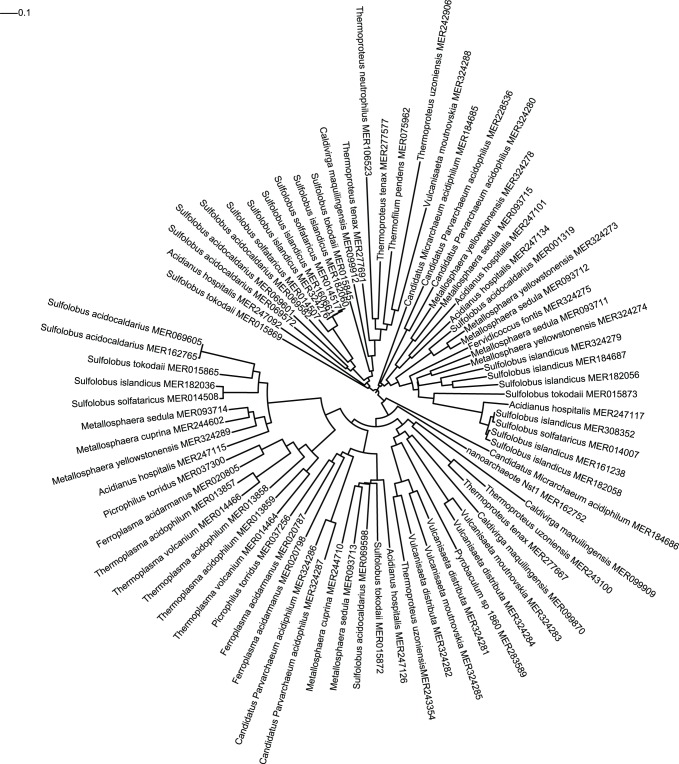
A Neighbour-joining tree for the thermopsin family A5. The tree was generated from the alignment of peptidase domains using FastTree. Labels are species names and sequence identifiers from the MEROPS database. Classification is indicated by coloured dots: yellow = Sulfolobales; red = Euryarchaeota; dark blue = Nanoarchaeota; light blue = Thermoproteales; green = Fervicoccales. The probable root is indicated by a black dot.

A phylogenetic tree was constructed from an alignment of the amino acid sequences of the thermopsin homologues. This is shown as a circular cladogram in Fig. 2. The tree is unrooted. It should be remembered that this tree is at the protein family level, and contains more than one protein species. For example, seven sequences are shown for *Sulfolobus acidocaldarius*. The tips are labelled with the MEROPS sequence identifier, and thermopsin is MER001319. The other *S. acidocaldarius* sequences are MER069582, MER69601 and MER069572, which cluster together and appear to be recent gene duplications within the *Sulfolobus* genus; MER06905 and MER162765, which are virtually identical; and MER069598. Thus there are four separate clusters of homologues from *S. acidocaldarius* which means that the tree encompasses at least four protein species (as defined by Barrett & Rawlings, 2007 [24]). There are 22 tips in a cluster which can be considered to be orthologues of thermopsin from different species, from MER184685 (from *Candidatus Micrarchaeum acidiphilum*) to MER182058 (from *Sulfolobus islandicus*).

This tree confirms the results obtained by examining the taxonomy of the species with homologues, because all the sequences from euryarchaeotes are neighbours, in two separate clusters (MER037300–MER014464, and MER037256–MER020798), both of which contain homologues from species in the order Sulfolobales. Both clusters are derived from a common node. The close relationship between sequences from members of the Sulfolobales and the Euryarchaeota also implies a horizontal gene transfer. The existence of two clusters each of which contains proteins from the same species, implies a gene duplication almost simultaneous with the lateral gene transfer.

The single nanoarchaeote sequence (MER162752) is most closely related to sequences from members of the order Thermoproteales, again suggesting a horizontal gene transfer. Thermopsin itself is restricted to the orders Sulfolobales and Fervicoccales (the cluster from MER184685 to MER182058).

It should be noted that this evolution schema may well change as more archaean genomes are sequenced. It is also dependent upon the classification of archaean species accurately mirroring their evolution, and that the establishment of clades at the same taxonomic level also accurately mirrors the major evolutionary changes with the superkingdom.

The evolution of the thermopsin family suggests that lateral gene transfers between archaea are possible.

## Supporting Information

Figure S1 **Alignment of peptidase domains from the thermopsin family A5.** Sequences were aligned with ClustalW and are displayed using Chroma [25]. The alignment is numbered according to preprothermopsin. Potential active site residues mentioned in the text are asterisked.
(DOCX)
