# Supplementary material for: Evolution of the Thermopsin Peptidase Family (A5)
Source: PLoS One. 2013 Nov 27;8(11):e78998. doi: 10.1371/journal.pone.0078998 (PMC3842238; doi:10.1371/journal.pone.0078998)
Supplement: Figure S1 — Alignment of peptidase domains from the thermopsin family A5. Sequences were aligned with ClustalW and are displayed using Chroma [25]. The alignment is numbered according to preprothermopsin. Potential active site residues mentioned in the text are asterisked. (DOCX) [file pone.0078998.s001.docx]

5 6 7 8 9 10 11 12

234567890123abc45abc67890123456ab7890123456789012345678901ab23456abc789012345678901234a567a890123

* *

MER001319/32-330 (9)YVNPYLYYTSPP---A**P**---**AGIA**S**F**GLYNY--SGNVTPYV**I**T**TN**E**MLG**Y**VNI**T**SL**L**A**--YNREA---LRYGVDPY**SA**T**LQ**F**N**I**VL**-SVN-TSNGV**Y**

MER324289/76-250 --------------------------------------------------------------------------------**ASLQLNAML**---------NG

MER093714/51-249 ------------------------------------------------Y**VMG**V**MNL**S**SL**QI----GRSYI-SGQPFEYG**NASLQLNAML**---------NG

MER244602/49-247 ------------------------------------------------F**VEG**V**VNL**T**SL**SI----GRSFI-SGQLFQYG**NASLQLNVML**---------NG

MER247115/50-249 ------------------------------------------------Y**VIG**T**VNI**T**SL**NI----GNSYLSSGMFFTKG**NASLQLNAMI**---------DG

MER182036/47-250 -----------------------------------------------**D**F**VMG**K**INI**SY**L**NI----GSSYLPGGEYFTTG**NASLQLNAMV**---------LG

MER014508/48-251 -----------------------------------------------**D**F**VMG**R**INI**SY**L**NI----GSSYLPGGEYFTTG**NASLQLNAMV**---------LG

MER015865/49-249 -------------------------------------------------**VMG**V**INI**T**SM**NI----GSSYLPNGQYLTTG**NASLQLNAMI**---------DG

MER069605/49-250 -------------------------------------------------**VLG**E**INI**S**SL**YI----GNSYLSNGQYLTTG**NASLQLNAMI**---------NG

MER242906/105-339 -------------------**P**---**LGVV**Y**Y**GLAVV--GGRPLPAP**Y**E**G**GA**VKG**C**FSL**R**GF**R**A**YSLSPLG--------RAA**AYSIQLNAYV**------EAGGG

MER037256/35-313 ---QVNPGYFIKGEP---A**P**---**MGIS**D**L**GLDSG------SPFY**L**N**TS**S**FMG**S**ITI**Y**N**ATFYNYTSKS----------T**SFSIQLNT**NF---FIFGSKT**Y**

MER020787/67-360 ---SVNPENLYRNEP---A**P**---**VG**M**A**D**Y**GLGTETLLGGYTPYE**Y**N**TT**S**FLG**S**AKI**Y**NL**S**V**ANSSSGN----------K**CMSVQ**F**N**IN**L**--VFNNSNNK**Y**

MER020798/40-323 ---VVYPEGYYTKEP---A**P**---**MGIA**D**Y**GIGPGG-----TPSS**F**NS**T**A**F**T**G**I**VNI**T**DL**K**T**YNASISS--------CPN**NM**G**IQLN**L**I**Y--SFQNGNST**Y**

MER244710/76-347 -------------EP---AQ---**MGIA**D**F**GIGPN------GPCI**L**T**TT**Q**FEG**C**V**L**I**N**NL**Q**S**VTSTSQG--------TSQ**CVS**F**QLNVVL**--NYDNQGSQ**Y**

MER093713/1-275 -----------------------**MGIT**D**Y**GVGPN------GFYS**Y**D**TT**Q**FLG**T**V**Y**I**N**SL**Q**A**LTFTSGS----------T**AV**TF**QLNVML**--NYEARGSS**Y**

MER069598/61-341 ---YISVYSLHYSEP---A**P**---**MGIT**D**Y**GLSPN------GSYI**L**T**TT**Q**WMG**V**INL**Y**GL**S**T**NT---------------T**SVS**F**QLN**LN**L**--HYYLNGYT**Y**

MER015872/47-337 ---YVNINAFYSSEP---A**P**---**MGIA**D**Y**GIGPN------GPYV**L**T**TT**Q**FLG**Y**INI**I**DL**S**A**QTFNGTQ-------LVNN**CVS**F**QLNAVL**--TYNHNGIT**Y**

MER247126/53-333 ----VNVYSLYNSEP---A**P**---**MGIA**D**Y**GIGPN------GPYIRN**TS**Q**VL**AK**AHI**C**SL**N**V**DSSINTD-----------**CVS**A**QLNVVL**--SYCYNGNQ**Y**

MER277667/42-293 ------PPSWYSGDN---A**P**---**VGIT**D**Y**GVYQN------VPYS**Y**S**TN**E**FMG**N**FTI**Y**S**AYFDSNSSSY---------PH**SFSIQLNVVL**--NYTSGGTP**R**

MER243100/6-269 ------PPSWYSGSQ---A**P**---**VGVT**D**Y**GAYYS--GSAEIPYE**Y**S**TN**E**FMG**N**FTI**Y**T**ASFTSNSKFY---------PH**SFSIQLNVVL**-NYTKTSGQSA

MER324286/35-305 (7)GVNLYACKYVEP---A**P**---**MGLV**D**Y**GVSSYG-----NVYN**I**S**TH**S**IE**SS**INL**Y**NF**V**T**NFSNDYN------------**ASIQLN**LN**V**--MLKYENKT**Q**

MER324287/43-322 (7)GVNLYSCRYSEP---S**P**---**MGIV**D**Y**GVTSYG-----NIYN**L**S**TN**S**IK**AS**IT**DE**GM**L**T**NISGNYN------------**ASIQLNS**N**V**--LLSYGNLS**Q**

MER037300/1-287 (9)DGVIEPSYNNAP---A**P**---**FGIG**F**Y**GTENI--NGRLTGFN**L**T**T**PG**IM**AS**IKI**Y**NM**S**D**FYLLNDG---------PE**S**ETF**QLNAVL**SNVTLFGKSG**Y**

MER020805/78-357 ---NGVIAPSYQSAP---A**P**---**MGIG**F**Y**GTQNE--SGKLVGYN**L**T**T**PS**VM**AS**I**G**I**Q**NM**SQFYLLNDG---------PT**S**ETF**QLNSVL**TNVTLFGNST**Y**

MER013858/88-366 ---GNTVSPTYATTP---A**P**---**MGIG**D**I**GLKYL--NGQTLPYE**I**N**FT**S**TE**AS**VTI**D**NL**S**D**FYLLNDG---------PH**SV**T**IQLNSVL**TNVSILGNSS**Y**

MER013857/71-370 ---NGTVQPLYTQSP---A**P**---**MGIG**F**F**GLENI--SGQLVGSN**Y**Y**AS**S**F**AAT**INI**T**NL**S**V**FNLADDA---------PS**SM**TF**QLNTV**TANTTLFGNSS**Y**

MER014466/86-385 ---NGVVELLYTQSP---A**P**---**MGIG**F**F**GLENK--NGQLMGSN**Y**Y**A**YS**F**SAT**VNL**T**NL**S**V**FNLANDG---------PN**SISIQLNTVL**DNTTILGTSN**Y**

MER013859/74-368 ---DGHITLTYDSAP---A**P**---**MGVA**DVGLFNS--SGSITPYS**V**N**TT**G**IMG**A**VDL**N**S**TQ**V**FYLYDDQ---------PY**SF**A**VQLNAV**AHDVDLFGNSS**Y**

MER014464/71-365 ---DGIITPTKIASP---S**P**---**MGLS**D**I**GLYNN--SGHLSAYN**V**TS**S**S**VMG**E**ITL**N**S**SNEYYLDDDM---------PN**SFSIQLNAVL**NNVTLHGTPK**Y**

MER324284/152-397 ------------------A**P**---**IGIV**D**Y**GLMPS--QVGYIPYS**Y**M**TN**E**FLG**E**ARV**L**SI**G**V**QPLTNCP------PLPPG**SFSLQLNAVL**--EMVVNNKT**Q**

MER324283/145-398 ----------HYSTPFINA**P**---**IGIV**D**Y**GLAPS--QLGYIPYS**Y**T**T**GE**FIG**E**AKI**M**S**AS**V**QSLTNCS------SLPPN**SFSLQLNAVL**--EMMINNQT**Q**

MER099870/119-365 -------------------**P**---**IGVA**D**Y**GVALYY-GEPLLAYS**Y**E**TS**E**VLG**V**A**F**I**R**SL**N**V**TDVRGDC----GVKAGDDY**A**D**IQLNSIV**------KAGDG

MER324282/174-453 -------------PN---F**P**---**MGIA**D**Y**GLALL--RGNLLAYE**Y**S**TD**M**F**V**G**E**VTI**Y**N**AS**T**--TEPSYCPSLEVKPGNTW**FS**T**QLNVV**M-LIQTANGNT**Q**

MER324281/175-455 -------------------**P**---**MGIA**D**Y**GIMKV--GNEYMAYS**Y**K**TN**E**F**V**G**E**TTI**Y**N**AI**T**--QEPNSCLTGSVEPGNDW**FSIQLNTIL**-IVNTTSGNA**Q**

MER324285/177-456 -------------------**P**---**IGIA**D**Y**GIAII--NGTYFAYE**Y**E**TN**E**FIG**K**ASI**Y**N**VF**T**--QEPSSCPTGYVELGNNW**FSIQLNTVL**-VVNTLSGYT**Q**

MER243354/173-432 ------------------------**G**A**A**D**Y**GIGLA--GGRLVAYS**Y**S**TD**M**F**V**G**R**V**A**V**Y**G**AD**T**--EEPGYCPTLSAEPGSRW**FSVQLNV**EM-EVRTADGGT**Q**

MER283589/126-394 -------------------**P**---**IGVS**D**Y**GVVFTN-PP--VVYN**Y**T**TR**E**FR**LR**V**VWL**G**GE**A**ASPRGDA------------**YSIQLNVY**A-EVN-TSKGI**H**

MER247134/247-532 -------------------**P**---**TGIA**S**F**GVSNT------SPYE**I**K**FS**S**V**A**G**Y**FNI**S**CI**L**A**YNSSQNL-------VKPCE**ASLQLNAVL**-VVCNENNAT**Q**

MER247101/203-469 ----FINPYLKLSTP---S**S**---E**GIA**S**Y**GILNET-KTT-KPYL**I**K**TT**S**ILG**K**FNI**S**CI**L**A**YNYSQNL-------VPPC**SASLQLNVVL**------KTCS**Q**

MER093715/210-491 -------------------**P**---**MGVA**S**Y**GLFNR--SGVPVPYV**V**K**AS**S**V**V**G**F**ANI**S**SI**F**A**--YNQTA---EKLNVSPY**SASLQLNV**P**L**--VVINGKQN**Q**

MER324278/228-482 -------------------**P**---**MGIA**S**Y**GIYNR--SGTPVPYT**V**R**A**PV**VLG**W**VNV**S**SI**L**A**--HDSSA---RILNVSPY**SASLQLNV**P**L**--VVYTGQGN**Q**

MER324280/303-587 (8)FVSYNQTNSSLP---S**P**---**TGLA**S**Y**GLTEN--GNLISPYC**L**K**TN**S**LLG**L**ASI**T**NI**S**S**VYLAPIR------NVSAA**SASLQLNGVL**-VVNDANGNS**F**

MER228536/300-582 (8)SISYNQTNASLP---A**P**---**TGLA**S**Y**GLD----GNNGNPYC**I**K**TS**G**LLG**L**VNI**K**NI**S**S**FALNPPK------DGGTSY**ASLQLNGML**-YVTNNNGVL**F**

MER184685/337-630 -------------------**P**---**TGIA**S**Y**GLYDTL-NKSTRTYQ**I**R**TN**E**IIG**I**ANV**S**SI**K**A**--YNATP---PS-NVSKY**GASLQLNVV**M-NGYNSNGKE**M**

MER015845/113-385 ---YTIPYNVYALIT---Q**P**(5)**IGIA**A**Y**GVSNK--SS----CV**I**T**TN**A**ILG**Y**FNI**S**SI**Y**A**--YNSTF---YM----HY**GASLQLNAVL**-R-----GGN**Q**

MER014507/10-308 ------LLLLLTPLVAISF**P**---**TGVV**A**Y**N------------GP**I**C**TN**E**VLG**Y**ANI**S**SL**L**A**--YNTSA---SQLGVPPY**GASLQLNVML**-EVN-TSGGE**Y**

MER182060/17-315 ------LLLLPTPLLAISL**P**---**TGVV**A**Y**D------------GP**I**F**TN**Q**VLG**Y**VNI**T**SL**Q**A**--YNASG---SKFGVPPY**GASLQLNVML**-QVN-TSNEE**Y**

MER014517/26-324 ------LLLLPTPLLAISL**P**---**TGVV**A**Y**D------------GP**I**F**TN**Q**VLG**Y**VNI**T**SL**Q**A**--YNASG---SKFGVPPY**GASLQLNVML**-QVN-TSNEE**Y**

MER324276/33-291 --------------------------------------------**I**F**TN**Q**VLG**Y**VNI**T**SL**Q**A**--YNASG---SKFGVPPY**GASLQLNVML**-QVN-TSNEE**Y**

MER069582/14-314 -------LLNPLFLVVHSL**P**---**TGVS**A**H**D------------GP**I**Y**TN**A**VLG**Y**ANI**T**SL**Q**A**--YNSSF-------NVPY**GASLQLNVVL**-EATSTNGNT**Y**

MER069572/8-307 ---ILYLLTCALCVHFSAV**P**---**IGIS**S**Y**Q------------GT**I**L**T**PS**VLG**Y**ANI**S**SL**L**A**--YSNSS-------QNPY**GASLQLNVML**-QVNTTTSKT**Y**

MER069601/12-309 ----LLLTLLPVIGFAVTA**P**---**VGIS**S**Y**S------------PT**V**T**TT**S**ILG**Y**ANI**S**SL**L**A**--YNSTF-------FQPY**GASLQLNAIL**-EVD-TPSNT**Y**

MER182056/124-400 ---PVDIHYERSSLP---A**P**---**VGIV**D**Y**GVVNS--TGRLIGEI**L**K**YN**E**AIG**Y**ITI**Y**NI**S**A**--YNSTF---EI----PS**GA**G**LQLNAVL**-QVN-TLTKA**Y**

MER184687/128-406 ---PVSVYYYKSSLP---A**P**---**IGIT**D**Y**GVYNV--SGILKGSI**V**K**Y**LE**AIG**Y**VTV**Y**NI**S**A**--FNATP---PM-GTNRS**GASLQLNVVL**-QVN-TNNES**Y**

MER324279/124-393 ----VDLYYIRQSLP---A**P**---**IGIV**Y**Y**GEYNG--SNFVNYTT**V**K**YE**E**AIG**Y**ATL**Y**SI**S**A**--YNSTP---PA-QVNPY**GASLQLNVML**-QIN-TEMSS**Y**

MER324274/148-427 ---PFQAH---SSPP---A**P**---**IGIA**D**Y**GVMNT--TSGLKGYI**L**N**FD**E**V**V**G**H**VKI**NK**I**G**A**--YNATP---PS-GINPY**GASLQLNVVL**-QVN-TPEGG**Y**

MER093711/139-414 ---PFTVH---SYLP---A**P**---**VGIA**D**Y**GVKNT--SKGLQGYI**V**K**FN**E**V**V**G**E**FTV**N**SI**S**A**--YNSTP---PS-GISPY**SA**T**LQLNVVL**-QVN-TVHGG**Y**

MER324275/142-417 STIPIDVYSYYNSLP---A**P**---**IGIA**D**Y**GVVNS--SGVLIPYE**L**L**YK**E**AIG**I**ANI**Y**SI**E**A**--YNSTS---PS-GVSEY**GASLQLNVVL**-QVN-TTYGS**Y**

MER014007/112-404 ---PVTLNYYILTRP---L**P**---**TGIA**D**Y**GLKIN--NGVISPYIEK**IK**S**VIG**A**VEI**NK**L**L**A**--YNSTP---PA-GVSQY**SASIQLNVVL**-QVN-TIGGS**Q**

MER308352/118-388 ---------YILTRP---L**P**---**TGIA**D**Y**GLKIN--NGVISPYIEK**IK**S**VIG**A**VEI**NK**L**L**A**--YNSTP---PA-GISQY**SASIQLNVVL**-QVN-TIGGS**Q**

MER182058/114-405 ----VTLNYYILSRP---L**P**---**TGIA**D**Y**GLKVN--NSVISPYIEK**IK**S**VIG**A**VEI**NK**L**L**A**--YNLTP---PA-GISQY**SASIQLNVVL**-QVN-TIGGS**Q**

MER247117/114-407 ----VTLNYYVLTRP---L**P**---**TGIA**D**Y**GLKVS--NGTVSPYIEK**IK**S**VIG**A**VEI**NK**L**L**A**--YNSTP---PS-GIGQY**CASIQLNVVL**-QVN-TISGT**R**

MER015873/97-383 ---VDVQLYYGSGRP---A**P**---**TGIA**D**Y**GIELN--GGTVKPYVEE**FN**G**VIG**V**AQI**Y**SI**S**A**--LNGTN----------Y**GASLQLNTVL**-QVN-TAQGT**Q**

MER093712/24-320 ---YVNPFLYYTSPP---A**P**---S**GIA**S**F**GLYNN--SGKVTPYV**I**E**TS**K**VLG**Y**ANI**T**SL**L**A**--YYKQA---RKYGVNPY**SA**T**LQ**M**NVVL**-QVN-TTQGT**F**

MER324273/36-334 (8)YVNPYLYYSSPP---A**P**---**GGIA**S**F**GLFNY--SGKVTPYI**I**T**TN**K**VLG**Y**TNV**S**NL**L**A**--YYKMA---RKYGVNPY**SA**T**LQ**M**NVVL**-QVN-TTHGT**F**

MER277577/121-378 ------------------L**P**---**TGIV**S**Y**-----------PQSP**V**E**TN**A**V**A**G**F**FNI**T**DI**S**A**----------------ASY**A**T**LQL**S**VIV**-QADLVGGGV**Q**

MER277691/117-380 ------------------L**P**---**VGIA**S**Y**-----------PAEL**L**S**TQ**E**V**A**G**F**FNI**T**SI**S**A**--YNPNF-------NPSD**GASLQLNAVV**-QVRLADGST**Q**

MER075962/125-409 ------------------L**P**---**AGLA**S**Y**-----------PDVA**V**N**TS**M**VLG**F**FNV**S**A**AS**A**KSYSSRA-------AEAD**AWSLQLNAVV**-EVSLAGGRK**Q**

MER099912/105-397 ----IVNAGINVIGL---A**P**---**TGLS**S**I**-------------IP**I**N**TT**A**VLG**Y**FNI**SI**I**R**A**--WNASYTAVNVLRVPKS**SASLQLNAVV**-RVELINGSF**Q**

MER015869/23-300 ---IGFIPRYNASSQ---Y**A**---**MGVS**S**I**G---------SSSLS**I**S**TN**E**VLG**Y**VEV**F**SL**S**S**----------------SS**PFSLQ**F**NAVI**-KATNIYGEI**L**

MER247092/144-398 ----------------LAS**P**---R**GIV**S**Y**GIYDY--AGELQNYC**I**R**TN**S**ILG**Y**FNI**S**CF**E**P**---------------KGCL**ASLQLNAVL**---LINGGND**Y**

MER184686/77-307 --------------------------------------------**I**Y**TK**S**VRG**I**ANI**SQ**L**N**A**VSSIGYN---------HS**G**S**SLQLNAVL**--YTCTNDIQN

MER099909/26-283 -----------------------**VGVS**DNGTAELW-NGSIIHYS**Y**H**VN**A**VMG**Y**LTL**Y**N**ASFTIISLAA--------SGN**VASIQLN**L**VV**------TNGI**E**

MER324288/158-372 --------------------------------------------**I**T**TD**A**VLG**F**FNI**T**S**ANIENSTGAA---------AP**GFSLQLNAYV**--IIEYGDGE**E**

MER106523/120-387 ------------------L**T**---**TGVV**S**Y**-----------PRGE**V**R**TR**W**VMG**Y**FNI**S**DI**N**T**---------------RSG**SW**A**V**F**L**GLP**V**-EVRLVNGTAV

Consensus/80% ...................s...hGls.b...............h.hp.hbG.hpl.sb.s..................shSlQLNshl..........b

13 14 15 16 17 18 19 20

45678901abc2345678901abc234567890a12345678901q23a45678901234abc5678901abcdef23a4567abc8901234567a890

** *

MER001319/32-330 A**YWLQDVG**---Q**F**Q**T**N**KN**S**L**T---**FIDNVWN**L**T**-**G**SL**S**T**LSS**SAI-T**G**-**NG**Q**V**ASAGGGQ---TF**YY**DVG------PS-**Y**T**YS**---**FPLSY**IY**IIN**-MSY

MER324289/76-250 S**FWAQDVM**---L**F**HQT**SN**D**T**FTVT**LV**I**N**F**WN**L**T**-**G**PFTA**L**H**S**NT--TTF**R**NL---------GVYC**YQ**GPT---------**F**N**VT**---**LP**V**SL**A**LFMN**SSGG

MER093714/51-249 T**YWAQDVM**---L**F**HEI**NN**R**T**FQVY**MV**I**N**F**WN**L**T**-**G**PFVS**LV**QNT--TTF**DG**L---------GVYC**YQ**GPT---------**F**N**IT**---**LP**V**SL**S**LFMN**SSQH

MER244602/49-247 T**YWVQNVM**---L**F**HEL**SD**K**T**FEVI**MVVN**F**WN**L**T**-**G**PFTT**L**IQNS--TIF**QG**L---------GVYC**YQ**GPR---------**F**N**VS**---**LP**V**SL**S**LFLN**SSRS

MER247115/50-249 K**YWAQDVA**---L**F**HEI**NN**KEFEIT**MI**I**NLWN**L**S**-**G**PFKI**L**K**N**NV--TTY**QG**L---------GVYL**YQ**GPT---------**F**N**VT**---**LPLNF**S**LFMN**ATNN

MER182036/47-250 K**YWAQNVI**---L**F**HQI**SN**N**T**FYAT**LIVNLWN**L**S**-**G**PF**S**NT**TG**NS--LVY**QG**L---------GVIC**YQ**GPT---------**F**K**VN**---**LPLSI**S**LFME**IVNS

MER014508/48-251 E**YWAQNVI**---L**F**HQI**SN**N**T**FYAT**LIVNLWN**L**S**-**G**PF**S**NT**TS**NS--LVY**QG**L---------GVIC**YQ**GPT---------**F**K**VT**---**LPLSI**S**LFME**IVNS

MER015865/49-249 L**YWAQDVI**---L**F**HQI**SN**NEFKAT**LV**L**NLWN**L**T**-**G**PFTIP**VN**GSV-TTY**QG**L---------GVIC**YQ**GPS---------**F**I**VT**---**LP**T**SI**V**LFM**IDNST

MER069605/49-250 I**YWAQDVI**---L**F**SQI**NK**T**A**FNAS**LVVNVWN**LL-**G**PFNLNL**S**KGIETTY**Q**NL---------GVIL**Y**TGPS---------**Y**I**VK**---**TP**VQ**I**K**LFM**IINST

MER242906/105-339 L**YWVQA**L**V**---R**Y**R**D**G**S**YE-----**FLDNVWN**M**T**-**G**PV**S**T**L**---------**SG**IAGFGSTTFLGRDE**YY**FY----------**V**SEL---P**PL**E**A**AC**L**E--IRT

MER037256/35-313 D**YWIQDVA**---Y**I**N**T**T**DN**C**I**A---**FIDNVWN**Y**S**-**S**RN**A**S**L**HFNSL-S**G**-**NG**T**I**ADHGRF------**YY**YCAPQDFPGNN-**I**D**LK**---**YP**AR**I**S**LMVN**-DTE

MER020787/67-360 V**YWVQDVA**---F**I**N**T**S**SR**A**I**T---**FIDNIWN**M**T**-**S**SG**A**S**M**Y**N**STV-N**G**-A**G**K**V**GNYSDSGY----**YY**SIASCTLPGND-**I**K**L**P---N**P**A**TI**NF**MVN**-STM

MER020798/40-323 Y**YWVQNVA**---V**L**N**T**S**NR**Q**V**V---**FIDNVWN**F**T**-**S**TN**A**E**M**H**N**STM-H**G**-**NG**T**L**MNSSSAHL----**YY**FASDNG------**T**FR**S**---**F**S**YS**NIK**L**R**S**VSYT

MER244710/76-347 AL**WVQDVA**---F**Y**N**T**Q**TN**Q**I**N---**F**E**NNIWN**F**T**-**S**PG**A**NV**TA**V---S**G**-**NG**S**L**YPSGSTK-----**FY**AYGPGSIQGNF-**I**T**LS**---**LP**SK**F**Y**LLVN**VSTN

MER093713/1-275 AL**WVQDVA**---C**F**N**T**V**NH**E**I**Y---**FIDNIWN**S**T**-**V**PF**G**NV**TG**L---Q**G**-**NG**QYAVSSSNGNYPSQ**VF**YGDVSHDPGSI-**V**TQ**S**---**LP**T**AF**D**LLVN**VSTN

MER069598/61-341 DL**WVQDVA**---F**F**D**T**Q**DN**N**I**Q---**ILDNIWN**F**S**-**S**PH**A**Y**ITS**V---Q**G**-**NG**N**I**YSYSKLNTT---**YY**AYEASGYPGSP-**A**T**LT**---**LP**A**TV**Y**LLVN**VSTN

MER015872/47-337 SL**WVQN**I**V**---R**F**D**T**A**NN**E**V**A---**FLDNIWN**Y**T**-QIY**A**NA**SG**L---S**G**-**NG**Q**I**GIVYYGSHAVEY**YY**DWA-NNYPGSF-**V**T**VT**---**LP**T**TI**L**VLVN**VSVN

MER247126/53-333 SI**WLQDVA**---L**I**MLF**NN**T**I**K---**FIDNIWN**L**S**-**S**VN**A**NVI**G**V---K**G**-**KG**T**L**CTYHGVT-----**FY**YYCANGYPGSP-**C**V**FS**---**YPLNF**CM**LIN**VSEN

MER277667/42-293 YL**WVQDVA**---V**I**N**T**A**TN**Q**L**T---**IVDNIWN**I**T**-**T**SN**A**Q**LN**RKLI-S**G**-**NG**K**V**YTYKGYT-----**YY**AYQYPYA-----**I**T**YS**---**YPLSV**S**LFVT**-VQL

MER243100/6-269 YL**WVQDVA**---V**I**N**T**A**TN**Q**M**N---**IVDNIWN**L**T**-**S**SK**S**V**L**K**G**L---S**G**-**NG**K**V**YTYQGQN-----**YY**AYQYPST-----**I**S**FT**---**YPL**Q**V**S**LFVT**VSIN

MER324286/35-305 A**Y**F**AQNVI**---L**M**N**T**K**TK**Q**I**N---**FIDNVWN**D**S**-**G**IN**A**S**LNS**TLI-E**G**-**NG**T**V**SPSNKNNT----**YY**FYQAENQTGDN-**I**T**LK**---NN**QTV**Y**L**R**TN**SSIS

MER324287/43-322 T**Y**F**LQNVI**---L**I**D**S**I**TN**S**I**A---**FIDNVWN**S**S**-**G**IN**S**S**MN**QKLI-V**G**-**NG**T**V**APSTKNES----**YY**FYEDTNQTGDN-**I**T**LR**---NN**Q**Q**I**Y**LMTN**SSIN

MER037300/1-287 S**FWTQNVA**---F**Y**S**A**R**TH**Q**L**Q---**FLVNIWN**F**S**-**S**PAIN**FTA**NSISYHS**SG**F**V**CAPTFYYA-------------VGPT-**I**N**VT**---**APFTL**N**LYMN**-SGI

MER020805/78-357 T**FWTQNVA**---F**Y**SER**TQ**T**I**Q---**FLTNIWN**F**S**-**S**PAVT**ISS**NVF-H**S**D**DG**I**L**CAPTFYYA-------------IGPT-**I**H**VT**---**TPFSL**N**LYLN**-STV

MER013858/88-366 S**FWTQNVV**---F**Y**S**A**R**TH**Q**I**T---**FIDNVWN**F**S**-**S**PAFN**MTT**NAL-H**G**-**NG**I**L**VPYVFYYD-------------IGPT-**F**N**VT**---**YPFTV**D**LYLN**-STV

MER013857/71-370 T**FWTQNVA**---S**Y**S**V**R**TH**E**I**S---**FVDNIWN**F**S**-**S**PT**A**V**MSS**NAI-LNS**TG**L**L**YPYSGVHIA------------LGPT-**F**Y**L**P---P**PFTL**T**LYLN**-TSE

MER014466/86-385 T**YWTQNVA**---F**Y**S**V**R**TH**E**L**E---**FIDNIWN**F**S**-**S**PT**A**V**MTP**NTI-LNS**TG**Q**V**LPYPGVHIA------------IGPT-**Y**Y**L**P---**TPFTL**T**LYLN**-TSE

MER013859/74-368 S**FWAQNVV**---I**Y**S**T**R**TH**Q**L**S---**FIDNVWN**F**S**-**S**PAFN**MTA**NAL-H**G**-**NG**K**L**VPGVFYYD-------------IGPT-**F**N**IS**---**FPFSL**K**LYLN**-SSL

MER014464/71-365 V**FWTQNVV**---V**Y**S**V**R**TH**E**L**T---**FIDNVWN**F**S**SRSSFA**LP**LSTL-E**G**-**KG**K**I**VSNKYYYY-------------VGPT-**L**N**IS**---**FPFTL**K**LYLN**-YSF

MER324284/152-397 Y**YWVQNVL**---I**I**D**P**TYGL**M**A---P**LVNVWN**M**S**-**S**AELF**MDP**LFI-V**G**-**RG**S**V**MNNE--------**VY**AYMGNW------**T**P**YE**---**MPLSV**N**LTI**ITNKT

MER324283/145-398 Y**YWVQDVL**---I**I**D**P**M**ND**L**M**A---P**LVNVWN**M**S**-**S**TELV**MNP**LFI-V**G**-**RG**S**V**MNNE--------**VY**AYMGNW------**V**Q**YE**---**APLTV**N**LTIT**SNKT

MER099870/119-365 Y**Y**I**VQDVV**---I**L**N**G**TAAS-----**IVDNVWN**I**T**-**S**LN**A**T**LSN**V---M**G**-L**G**S**I**GDFNGQQ-----**YY**AYS---------RV**I**GELK**TPFNL**T**LAI**MVSGV

MER324282/174-453 Y**YWLQDVL**---E**F**N**S**Q**SD**Q**F**Q---V**LDNIWN**D**T**-**G**ST**S**V**INS**ALI-S**G**-**NG**G**I**----TSSGSNVL**YY**DWG----IEQP-QS**VS**---**LPFTI**Y**LVIK**VGLN

MER324281/175-455 Y**YWLQDVV**---R**F**N**S**Q**SD**Q**F**Q---V**LDNIWN**Q**T**-**G**GS**S**I**LNS**ALI-N**G**-**NG**A**I**STSSTAVGNEQY**YY**DWG----IEQP-QS**VS**---**LPFTI**Y**LVIK**VGLN

MER324285/177-456 Y**YWLQN**I**L**---L**F**D**S**L**N**YT**T**Q---V**GDNVWN**S**T**-**S**IN**A**S**LSS**NLI-S**G**-**NG**V**V**SPVNTTVSN-VT**AY**AYY----IELT-QQ**AN**---**LPFTI**Y**LITR**TGLT

MER243354/173-432 Y**YWLQD**I**A**---S**Y**N**S**S**K**GI**V**E---**IWDNVWN**D**T**-ESS**A**A**LSP**GLI-A**G**-**SG**R**V**AGGFYSASS------------LRPP-RP**A**-----**PQ**Q**I**Y**LVVK**SGLS

MER283589/126-394 Y**YWLQNVI**---Q**F**--Y**N**GK**F**R---**ILNNIWN**H**T**-**D**AF**S**Y**LS**KEVI-K**G**-L**G**N**V**GNDRNPQGVLED**YY**YKI----AFDF-KD**A**A---P**PINT**S**LYIK**VGVR

MER247134/247-532 I**YW**P**QDVL**---L**F**L**T**N**ES**V**V**L---**YHDNV**L**N**L**T**-**N**PL**A**S**LSN**SSI-T**S**Q**NG**Y**V**MPTVNDGVTQYY**Y**GNYK----CAPY-**F**E**YN**---S**PFSG**L**LIMN**ESVE

MER247101/203-469 V**YWLQNVL**---E**F**L**T**L**KH**E**F**K---**LAD**D**I**L**N**I**T**-**C**ID**S**T**LSN**YSI-T**S**L**NG**Y**V**TMVNQSGKIEYY**Y**GNYY----CQSE-**L**K**YC**---**LPLCG**Y**LVTN**-VSL

MER093715/210-491 T**YWVQNVI**---V**F**M**T**N**ES**T**L**C---**Y**E**S**S**V**L**N**V**T**-**N**AN**A**T**LTN**ISI-Q**G**-**RG**G**V**YPPFNNG----I**YY**TYK------TKG**V**Q**YK**---**TPLSL**L**I**S**IN**VSVI

MER324278/228-482 T**YWVQNVA**---E**F**L**T**N**DS**V**L**C---**Y**Q**S**S**V**L**N**V**T**-**S**VN**A**T**LTN**NSI-T**G**-**RG**A**V**YPPFQNG----F**YY**TYI------TRN**Y**T**YH**---**LPLSL**E**L**S**VN**VSVL

MER324280/303-587 D**YWVQNVL**---Y**M**D**T**NF**N**D**Y**Y---N**T**ID**I**F**N**Y**T**G**S**SI**S**N**FTN**LSI-Q**G**-**NG**Y**V**YNDGNYTDFRQA**TQ**Y-----------**T**S**YS**---**LPFS**RT**IITR**SYLD

MER228536/300-582 N**YWLQN**T**M**---T**F**E**T**DYYYDY---N**TVNI**Y**N**E**T**G**S**FL**A**N**LTN**TSI-Q**G**-**NG**Y**V**YNSK--------**YY**AQSTSS------SQ**YS**---**LPIN**KVF**MTN**ISVE

MER184685/337-630 T**YWLQDVV**---R**F**N**T**S**DK**N**F**Y---**ILDNIWN**Y**S**-LPQ**A**N**MT**E--V-Y**G**-**NG**K**L**STYTFNSTYKQK**LY**VFSF---PKYY-**M**N**YS**---**LPLSI**K**LIT**I----

MER015845/113-385 SLF**LQNVI**---S**F**I**T**N**KN**I**L**Q---**FVTNIWN**L**T**-**S**PL**A**S**LNS**---------------------SF**FY**FNS----T-SY-ST**YR**---**LPFAG**Y**LIIN**-VSN

MER014507/10-308 Y**FWLQNVA**---D**F**I**T**N**ES**K**V**F---**FGDNIWN**S**T**-**T**PF**A**G**INN**--I-V**G**-**KG**E**I**YSTSDFF-SHSS**YY**AYG------TYY**I**K**YN**---**FPFSF**Y**LIIN**-ESY

MER182060/17-315 Y**FWLQNVA**---D**F**I**T**N**ES**K**M**F---**F**SE**NIWN**S**T**-**T**PL**A**G**INN**--V-I**G**-**KG**E**I**YSTSDLF-SHSS**YY**AYG------TYY**I**K**YD**---**FPFSF**Y**LIVN**-ESH

MER014517/26-324 Y**FWLQNVA**---D**F**I**T**N**ES**K**M**F---**F**SE**NIWN**S**T**-**T**PL**A**G**INN**--V-I**G**-**KG**E**I**YSTSDLF-SHSS**YY**AYG------TYY**I**K**YD**---**FPFSF**Y**LIVN**-ESH

MER324276/33-291 Y**FWLQNVA**---D**F**I**T**N**ES**K**M**F---**F**SE**NIWN**S**T**-**T**PL**A**G**INN**--V-I**G**-**KG**E**I**YSTSDLF-SHSS**YY**AYG------TYY**I**K**YD**---**FPFSF**Y**LIVN**-ESH

MER069582/14-314 Y**FWLQNVA**---Q**F**V**T**N**ES**L**M**C---**FTDNVWN**Y**T**-**T**AT**A**E**ISN**--V-T**G**-**NG**G**I**GFTYNILFGHPT**FY**GYS------TEP**M**P**YR**---**FPLAL**Y**LLIN**-ESL

MER069572/8-307 Y**FWLQNVA**---S**F**L**T**N**DK**V**A**Y---**FLDNVWN**V**T**-**T**PYTQ**ISN**--V-K**G**-**NG**Q**V**YTISNGP-YGQS**FY**GYT-----SNYP**I**R**YN**---**YPFSF**YM**FIN**-TSY

MER069601/12-309 Y**FWVQNVA**---G**F**I**T**S**NN**T**L**F---**F**N**DNIWN**A**T**-**G**MD**S**N**IT**E--V-I**G**-**DG**N**I**STCDSCQAPQTF**Y**GASS------QQT**I**Y**YH**---**FPLSF**YM**FIN**-VTP

MER182056/124-400 Q**FWLQNVV**---D**F**I**T**T**ND**T**Y**Y---**FTDNIWN**S**T**-**S**EF**S**N**MSN**GSV-S**G**-**NG**H**V**YFYNKEL-G--Y**FY**GVS----T-NN-**F**T**Y**V---**TPFSL**I**LYIK**LLSI

MER184687/128-406 Q**FWLQNVI**---D**F**I**T**SI**K**I**Y**Y---**I**E**DNIWN**F**T**-**S**NI**S**Y**LTN**ASV-S**G**-**KG**A**V**YYYKDGN----F**YY**AYS----T-TY-**Y**N**YT**---**FPFSL**I**LFTK**LDNI

MER324279/124-393 Q**FWLQNVI**---T**F**I**T**SG**D**Y**Y**C---**I**D**DNVWN**S**T**-LRP**S**L**LTN**SSV-T**G**-**NG**A**V**YFSDEQH-G--Y**YY**GYT----V-GG-**F**N**YT**---**FPFSV**I**LHIK**LQNV

MER324274/148-427 Q**YWLQDVL**---H**M**E**T**N**ES**M**Y**Y---VE**DNIWN**F**T**-**S**YP**S**T**LSN**SSV-K**G**-**RG**A**V**YNVASSS-SLQY**YY**AYG----S-PL-**Y**P**YH**---**LPLST**D**LLIK**-EEV

MER093711/139-414 Q**YWLQDVL**---L**F**F**T**N**ND**T**A**C---VE**DNVWN**F**T**-**S**YP**S**I**LSN**STL-T**G**-**QG**H**V**YPYREGN-YIEY**YY**AYG----I-SA-**F**R**YS**---**TPF**K**G**Q**LVMR**-TNV

MER324275/142-417 T**YWVQNV**P---N**F**W**T**N**NN**T**L**F---**FVDNVWN**F**T**-**S**HF**S**F**LTN**QSV-K**G**-**NG**Y**V**YVDNVGE-D---**YY**AYS----T-EM-RK**YT**---**LPLNL**K**LAVS**-ENF

MER014007/112-404 QL**WLQNVI**---Q**I**Y**T**N**ND**S**Y**I---**FLDNIWN**F**T**-**G**KI**S**I**LSN**STV-K**G**-**NG**I**V**YVTNNGN----D**YY**AYG----T-NF-ST**L**L---**IP**SLKY**LLIN**-TSY

MER308352/118-388 QL**WLQNVI**---Q**I**Y**T**N**ND**S**Y**R---**FLDNIWN**F**T**-**G**KI**S**I**LSN**STV-K**G**-**NG**I**V**YVTNNGN----D**YY**AYG----T-NF-ST**L**L---**IP**SLKY**LLIN**-TSY

MER182058/114-405 QI**WLQNVI**---Q**I**D**T**N**NN**F**Y**S---**FLDNIWN**F**T**-**G**KI**S**I**LSN**STV-K**G**-**NG**I**V**YVTNNGN----D**YY**AYG----T-NF-ST**L**L---**IP**SLKY**LLIN**-TSY

MER247117/114-407 QI**WLQNVI**---Q**I**N**T**Y**NN**S**Y**R---**FIDNIWN**F**T**-**S**YP**S**I**LCN**-CV-K**G**-**NG**G**V**YFMFQGI-RVCD**YY**AYS----T-NY-**F**T**L**F---S**P**SIEY**LIIN**-TSY

MER015873/97-383 Q**YWLQNVI**---Q**F**I**T**N**ES**I**Y**R---**YVDNVWN**F**T**-**S**SP**S**E**LNS**SLI-H**G**-**EG**Y**V**YTTGILS--PCS**YY**AYA----T-QY-**F**N**MT**---**YPF**Y**A**V**LFIS**-VQT

MER093712/24-320 A**YWLQDVG**---S**F**Q**T**N**TN**Q**V**T---**FIDNIWN**L**T**-**G**NP**S**T**LSS**SAV-S**G**-**NG**K**V**ASAGSGN---TF**YY**DVG------PT-**F**T**YS**---**FPFSY**VY**VVN**-TSY

MER324273/36-334 A**YWLQDVG**---S**F**Q**T**N**NN**K**V**T---**FIDNIWN**L**T**-**G**NP**S**V**LSS**SVV-S**G**-**NG**K**V**TSAGGGN---TF**YY**DVG------PT-**Y**T**Y**Y---**YPFSY**VY**VVN**-VSY

MER277577/121-378 Y**YWA**REM**L**---V**F**-**S**P**N**GQ**L**K---**F**E**N**Y**V**A**N**S**S**-**S**PG**S**S**M**Q**S**V---S**G**-**RG**H**L**SLSR---------**Y**LYS------SEWSP**YS**---**LPISG**F**LLIT**-AEA

MER277691/117-380 Y**YWVQD**I**A**---W**F**E**T**S**R**GQ**V**N---**FGDNVWN**S**T**-**S**PS**S**SV**SP**SSI-S**G**-**QG**S**V**NNG---------**VY**LYG------TYLQP**Y**A---**LPLAG**F**LAVR**-AYA

MER075962/125-409 Y**YWVQN**M**V**(7)S**Y**E**N**K**TE**K**A**IMYQV**WNNIWN**N**T**-**G**RL**S**L**LSD**ERI-S**G**-**CG**G**V**YRDRDEY-----**YY**ACV---------**Y**T**YS**PCD**LPLAG**F**LVVR**-AYA

MER099912/105-397 E**YWLQD**A**L**---I**F**I**T**S**N**GV**F**S---V**ADNVWN**A**T**-**A**PG**A**NV**SS**SLI-T**G**-L**G**R**V**YNAVGVS---QE**YY**GYV----G-NL-**T**R**YQ**---**LPLSG**Y**L**E**VN**-VTL

MER015869/23-300 D**FWVQN**I**I**---E**F**--N**KQ**E**L**N---**FTD**E**IWN**F**T**-YSY**A**S**INP**NLI-S**G**-**KG**E**V**YSTDING-HIVN**YY**SYS----T-PY-QS**YS**---**LPLTY**I**LFIS**-ISY

MER247092/144-398 E**Y**F**LQN**I**I**---K**F**E**N**CF**H**W**F**Y---**L**E**DNIWN**F**S**-**S**YR**A**N**I**YHV---E**G**-**KG**I**I**NSYCNRT-----**YY**CYSPPCLFD---**I**K**YS**---**LPLAG**Y**LIIN**VTTV

MER184686/77-307 V**YWLQNVV**---R**F**DQY**TS**K**L**E---**FA**E**NIWN**F**S**-YTY**G**N**M**QQQPLSNN------------------**Y**SKP---------**V**P**YE**---**LPLNI**S**VLIN**-ITT

MER099909/26-283 Y**FWVQN**I**A**---H**I**GEF**N**GSRYMVW**FWD**D**LWN**I**T**-RVN**A**L**LD**LSLI-K**G**---------NRTYPVTE**HY**AYYYRYVAPPV-**M**L**VK**---**APFTV**SC**MVT**-VNL

MER324288/158-372 I**HWVQD**A**L**---V**I**I**D**D**Q**YW**F**Q---GEMD**V**V**N**YIG**S**SRNF**I**YE-------**QG**KCVL------------------------**G**C**FE**---**TPMSG**V**LVI**A-VNS

MER106523/120-387 H**YWA**--**VV**---W**I**SKS**D**WEEG-----**A**E**V**YYY**S**-**S**RD**S**TPY-----Y**G**GY**G**YQYFPSAD------**YQ**VYRSEDADFDKV**V**S**FR**SPK**MPLAG**YF**VG**A-VGV

Consensus/80% .aWhQsVh....b.s.pp.h....bhsNlWN.*.s..t.bss.....t.pG.l............hb............h.hp...hPbsh.lhhp....

21 22 23 24 25 26 27 28

12345678901234567abcd890123456789012a345abc6789012345678abc9012abcde3a45678901234567890123456a789012

* *

MER001319/32-330 TSNA**V**Y**V**WI**GY**EIIQIG----QTEYGTVNY**YDKITI**-YQP---NIIS**A**S**LMING**NN---**YTP**N-----G-LYY**DAELVWGG**G**GNG**AP**T**S**F**N**S**L-**NC**T**LGL**

MER324289/76-250 ------**L**S**FGY**SLN-----------GDRKV**F**L**TL**P**F**----------S**G**Q**F**R**LGG**LS---**IAG**L--------PN**DLELVWGG**P**GGG**SV**V**D**M**SA--**VG**FAE**L**

MER093714/51-249 ------**L**Q**FGY**SIN-----------GVKRV**Y**L**TL**P**F**----------H**G**L**F**K**LGG**LS---**VNG**L--------PN**DLE**M**VWGG**P**DGG**SV**V**D**M**IA--Q**G**S**E**E**L**

MER244602/49-247 ------**L**Q**FGY**SVN-----------GERNV**Y**L**DL**P**F**----------S**G**L**F**K**LGG**LS---**ANG**L--------PN**DLEIVWGG**P**GGG**SS**V**D**M**TA--Q**G**R**E**E**L**

MER247115/50-249 ------**L**E**FGY**CIN-----------GKKYV**Y**QV**L**P**Y**----------F**G**N**F**Q**IGG**LS---**I**L**G**L--------PN**DLE**F**VWGG**P**GCG**SE**V**C**M**SG--EMS**E**EI

MER182036/47-250 T-----**L**D**FGY**NIN-----------EERGI**Y**F**R**YP**L**----------I**G**L**F**Q**LGG**LS---**L**L**G**L--------PN**DLELVWGG**P**GGG**SV**V**F**M**NV--**SS**VA**NL**

MER014508/48-251 T-----**L**N**FGY**NIN-----------GQKGI**Y**F**R**YP**I**----------I**G**L**F**Q**LGG**LS---**L**L**G**L--------PN**DLELVWGG**P**GGG**SV**V**F**M**NV--**SS**IA**NL**

MER015865/49-249 ------**L**Y**FGY**NID-----------NKSGI**F**Y**KI**P**L**----------S**G**E**F**E**IGG**LS---**IAG**I--------PN**DVE**F**VFGG**P**GGG**SV**V**D**M**QV--E**G**T**MN**I

MER069605/49-250 S-----**L**Y**FGY**YIN-----------GHSGI**F**Y**QL**P**L**----------T**G**S**F**R**IGG**FS---**A**I**G**L--------PN**DLELVFGG**P**GGG**SS**V**Q**L**I**T**--**TA**NA**NL**

MER242906/105-339 SGRY**V**E**L**AL-----------------NGTTM**DRI**E**L**--------TGP**A**R**IVV**E**P**Q----**VNA**R-----G-LPI**DLELV**V**GG**Y**GAG**MS**V**AEVADGE**A**E**LAL**

MER037256/35-313 INNK**P**A**V**N**FYY**FDS-----------NSWVK**YDTVIF**------NENGSYH**FMVNG**YN---**YNP**A-----G-LLS**DAELIIGG**P**GNG**SS**T**VDL**S**S-**N**IL**LSL**

MER020787/67-360 KNGS**P**E**V**EL**MY**NDG-----------YRWMT**YDS**P**VF**IFAT--NVTSDQS**FVVDG**YN---**Y**E**P**D-----GYSFY**DAELILGG**P**GDG**SS**T**VDM**S**S-**N**IQ**LG**I

MER020798/40-323 ASGY**P**H**I**YME**Y**NDG-----------NGWKT**YDTL**N**F**TFAS--SVSHNYG**FIVNG**QN---**L**L**P**N-----N-SYA**DA**G**LILGG**P**GN**STN**T**R**V**I**Q**A-**N**LT**MGL**

MER244710/76-347 AYGQ**P**V**I**Y**FWY**NDG-----------YGWIN**YDTVT**VTNAF---SSSN**V**Y**FLVDG**YQ---**YAG**N-----G-LYY**DAEL**D**M**V**G**P**GD**LTC**A**D**I**I**S**S-**NV**A**LNL**

MER093713/1-275 NLGQ**P**V**I**Y**FWY**NDG-----------YGWIN**YDTVT**VTNVQ---GATN**V**Y**F**E**VNG**YA---**LTG**N-----G-NNY**DAELVLAG**P**GGG**SN**T**Y**I**N**Q**A-**NV**D**L**L**L**

MER069598/61-341 SLGE**P**V**I**Y**FYY**NDG-----------YGWIL**YDTVT**VTNAQ---GSSQ**V**Y**FLVDG**FQ---**YNG**Y-----G-TFW**D**S**E**M**VFAG**P**GGG**ST**T**Y**V**Y**S**S-E**V**Y**L**E**L**

MER015872/47-337 SLGQ**P**V**I**N**FWY**NDG-----------YGWVK**YDTVV**VTNVE---GASN**V**K**FMING**NE---**YTG**W-----G-TFY**DAELVLGG**AY**GG**LN**A**Y**V**Y**S**A-**N**IY**MNL**

MER247126/53-333 SLGE**P**VAY**FWY**NDG-----------HGWVN**YDTVTF**LFPH----SRN**V**N**ITING**YQ---**YTG**S-----G-DFY**DIE**FD**I**V**G**P**GGG**TC**A**Y**F**N**N**A-**CV**Y**L**Y**L**

MER277667/42-293 VRGY**P**V**V**N**FY**FVYG-----------GKVVQ**YDSVTI**-----KVPSTS**A**S**YTV**E**G**YS---**L**L**P**S-----G-LYD**DAELV**T**GG**P**GGG**TS**A**MPL**T**Y-**NA**T**YTL**

MER243100/6-269 SQKY**P**V**I**N**FY**FVYG-----------GKVVQ**YDSVV**V-----KVPVSS**A**A**FYV**E**G**YT---**T**L**P**S-----G-LYE**DAELV**T**GG**P**GGG**SN**A**SPSAY-K**A**Y**YTL**

MER324286/35-305 RDGN**P**E**I**I**F**S**Y**NDG-----------KGWRE**YDKV**S**L**LVNN---SIKNYS**IIISG**FN---**YTP**------SYNFY**DA**G**IIIGG**P**GDG**YN**T**T**L**I**N**G-**SA**V**LTL**

MER324287/43-322 ANGS**P**E**I**I**F**S**Y**NDG-----------YGWKE**YDKVVF**NRLK---NVSN**A**S**MLISG**FN---**YTP**------GYNFY**DA**G**IIIGG**P**GAG**SN**T**T**M**I**N**G-**S**LL**LS**I

MER037300/1-287 VNND**S**A**V**F**F**N**Y**SVK-------TASMNVSGT**YDEVLF**NSTR---AVIN**P**E**YL**A**SG**TQ---**LTP**T-----GYIPY**DFEI**-----------------------

MER020805/78-357 VDRD**S**A**V**F**F**N**Y**SIT-------SDNHVQSGS**YD**YA**IF**-NST(7)TAKA**P**E**YL**A**SG**TQ---**ITP**T-----GYIPY**DFEI**MV**GG**P**GGG**ST**T**S**I**Y**N**V-**NA**T**MNL**

MER013858/88-366 INGN**S**A**V**Y**F**N**Y**SVF-------TSGKVYSGS**YDTVIF**-NST(5)RAPQ**P**E**Y**Q**IDG**YG---**YDA**T-----GYLIN**DAEV**M**IGG**P**GGG**ST**T**S**I**Y**S**I-**NA**T**M**H**L**

MER013857/71-370 INGN**N**VFY**F**N**Y**SI---------PTLGVSGT**YDRVTF**-NST(7)KAPP**S**Y**F**R**VSG**TQ---**LTP**L-----G-LLY**DAEI**M**IGG**P**GGG**ST**T**T**V**T**D**I-**NG**M**MDL**

MER014466/86-385 INGN**N**V**V**Y**F**N**Y**SI---------PQIGAEGT**YDRVTF**-NST(7)KAPY**S**Y**F**R**VSG**TQ---**LTP**L-----G-LLY**DAEI**M**IGG**P**GGG**ST**T**T**I**MGI-**NG**T**MSL**

MER013859/74-368 VGGN**D**A**I**Y**F**N**Y**SIE-------NGKITKSGS**YD**A**VTF**-NSS-GKASSP**A**R**FLISG**YS---P**SP**A-----G-LLY**DAEL**A**I**T**G**P**GGG**SN**V**N**V**YGI-**NG**T**MGL**

MER014464/71-365 ANGN**D**L**V**Y**F**N**Y**SIL-------NPSYSKSGS**YD**M**VTF**NSNS---SESG**A**Y**YFVSG**YV---**TSP**S-----G-LLN**DAELVIGG**P**GGG**SN**A**N**F**Y**S**I-**NG**T**MSL**

MER324284/152-397 VNGF**P**Q**V**LI**GY**SMG-----------GINTLV**DNVTF**LMTP----SWG**P**H**LVVNG**SE---**YSP**L-----G-YLI**DAE**F**VIGG**P**GCG**AM**V**R**V**N**E**L-**NA**T**LSL**

MER324283/145-398 IDGF**P**E**V**LL**GY**SMN-----------GVNTLV**DNVTF**LITP----SWG**P**Y**LVVNG**SE---**YSP**L-----G-YLI**DTE**F**VISG**P**GCG**AM**V**L**T**N**E**L-**NA**T**LSL**

MER099870/119-365 NSAH--**L**S**FGY**GVQ-----------GSLIW**FDNVTL**------INAGK**P**S**IIVNG**SS---**YV**EF-----G-IPI**DTELVL**T**G**P**VCG**IW**A**V**V**K**S**V-**NA**T**FTL**

MER324282/174-453 SNGY**P**WAA**FGY**----------SLNGQTTTW**YDNVTI**--MI---PSNY**A**S**ITVSP**PNPLLNKYV-------PFN**DAELV**V**AG**P**GD**SEC**T**I**A**N**T**L-**GV**E**LGL**

MER324281/175-455 SNGY**P**WAA**FGY**----------SLNGQTTTW**YDNVTI**--KI---TATS**A**Y**M**E**VSP**TK---D**G**RG-------LPI**DAELVIAG**PW**N**SEC**T**V**A**N**S**L-**DS**S**LSL**

MER324285/177-456 SNDY**P**W**I**A**FGY**----------SYNGRSITW**FDNVTI**--RV---KSSS**T**Y**M**E**VSP**IS---N**GG**G-------LMN**DAELVI**V**G**PW**N**SEC**T**A**A**R**S**L-**N**IT**LSL**

MER243354/173-432 AEGL**P**WAA**FGY**----------SLDGAHITW**YDNVTI**--RV---PATY**A**R**LVV**K**P**PG------N-------PLA**DAELV**V**AG**PW**S**SEC**T**Y**V**R**S**M-**SA**D**LAL**

MER283589/126-394 SDGR**P**WFA**FGY**DVG-----------RGVEW**YDNVTL**-NLA----ARS**V**A**IAV**R**G**LK---K**NP**R-----G-LELN**AE**G**V**V**GG**M**CC**REH**A**D**F**K**K**I-**SV**V**L**Q**L**

MER247134/247-532 KGQG**V**L**I**TMEVEVLQNG----TSSVMQSET**FDKILI**-HDP---DVKN**A**Y**FVVNG**KE---**YTP**A(4)SLG-SFY**DAELILGG**G**GNG**EI**T**T**F**E**K**L-**NG**L**LGL**

MER247101/203-469 EKCGIL**V**S**FA**VIFIKNG----SSTCYKEIV**FDNVLI**-----HGKFRF**A**Y**ILVCG**KH---**YTP**I-----G-SYY**DAELVFGG**G**GNG**EI**T**K**F**C**K**L-**NA**T**L**W**L**

MER093715/210-491 KKLG**V**R**I**G**F**D**Y**KVLENG----SVVNGSWNQ**FDS**P**LI**LDSG----VSQ**A**Y**LYVDG**YN----**SP**S-----TLNFY**DAELVFGG**G**GNG**EV**A**Y**F**Q**N**L-**SA**T**LA**I

MER324278/228-482 QGLG**V**Q**L**K**FW**VMVIQNG----SQYPMKGLT**FDSVL**V-KVP----AKK**A**F**LYVSG**YQ----**TP**V-----SINYY**DAELVFGG**G**GNG**AI**A**T**F**K**Q**L-**NA**Y**LAL**

MER324280/303-587 KGKG**V**W**L**Y**F**-**Y**DIPMNL(5)-TSRITNQTM**Y**A**KVFI**-YDP---YAVS**S**Y**FLVSG**YK---**T**LKN-----S-LYY**DAE**FT**LGG**G**ADG**ED**S**Y**F**Y**N**FT**SA**D**ISL**

MER228536/300-582 TGKGIF**V**Y**F**-**Y**ILPFNN(5)-TEQFTGKNI**Y**S**KVFI**-SDP---NVSS**A**Y**FLISG**YR---QL**N**N-----S-QYY**DAE**FT**FGG**G**GNG**EN**S**F**F**Y**N**TT**SA**D**IGL**

MER184685/337-630 -AKG**N**R**I**S**FGY**QILKND(16)NATPQSVIF**YDNVTI**---P---DLNNYS**ILVTP**YY---E**TP**GTINSNG-NYY**DAELIFGG**E**GNG**EN**T**T**F**S**S**M-**NA**T**L**Q**L**

MER015845/113-385 VSNG**V**K**I**S**FGY**IIIQNG----SIIEPMVRF**F**T**T**A**YF**--------PFK**G**Y**ILVDP**FN---**LTG**N-----Y-HAY**DTE**F**VFGG**YE**DG**EI**T**T**F**I**S**L-**NA**T**LAL**

MER014507/10-308 DTQG**V**Y**V**S**FGY**VILQNG----NISPPNPIF**YDTVFI**-PIQ---NLSF**A**S**IIIAN**Q----**TTP**S(7)YLG-NYL**DAELVWGG**F**GNG**ES**T**T**F**L**N**M-**SS**Y**LAL**

MER182060/17-315 NNQG**V**Y**V**S**FGY**VILQNG----NITPPNPTF**YDTVFI**-PVN---NLTS**A**S**IIIAN**Q----**TTP**N(7)YLG-SYL**DAELVWGG**F**GNG**AS**T**T**F**L**N**M-**SS**Y**LAL**

MER014517/26-324 NNQG**V**Y**V**S**FGY**VILQNG----NITPPNPTF**YDTVFI**-PVN---NLTS**A**S**IIIAN**Q----**TTP**N(7)YLG-SYL**DAELVWGG**F**GNG**AS**T**T**F**L**N**M-**SS**Y**LAL**

MER324276/33-291 NNQG**V**Y**V**S**FGY**VILQNG----NITPPNPTF**YDTVFI**-PVN---NLTS**A**S**IIIAN**Q----**TTP**N(7)YLG-SYL**DAELVWGG**F**GNG**AS**T**T**F**L**N**M-**SS**Y**LAL**

MER069582/14-314 SSNG**V**I**V**N**FIY**VVLQNG----SLVPPHPVT**YDSVFI**-PVP---DPQS**A**Y**IIVND**S----**LTP**S(13)LG-NLM**DTELVWGG**YE**NG**EF**T**T**F**T**Q**M-**SS**Y**LGL**

MER069572/8-307 SGSL**V**T**V**E**FGY**VIVQNS----TVIPPVVET**YDEV**Q**L**-RIN---GVQG**A**S**IIVND**S----**YTP**S(7)YLG-MLE**DCELVWGG**L**SNG**EK**T**S**F**E**N**M-**SS**L**LA**M

MER069601/12-309 TVRG**P**L**V**T**FGY**VILQNG----KITSPKVQV**YDN**A**II**-PVQ---GAIS**A**Q**IVV**Q**N**SYTYV**YN**QGLYSYYG-LKK**DVELVWGG**L**SNG**EH**T**T**F**K**E**M-**SS**L**LA**I

MER182056/124-400 NNNG**V**T**V**S**FGY**N---------NGSG--VIW**YDN**A**TI**-KVS---NVKS**A**Y**MLING**YN---**MTS**R-----L-QYY**DLELVFGG**IF**DG**EY**T**F**Y**K**S**M-**NA**S**LGL**

MER184687/128-406 SRQG**V**T**I**S**FGY**N---------NGTG--IQW**YDNVTI**-HVF---SVRS**A**Y**LLIDG**YN---**LTG**N-----Q-FYY**DLELVFAG**Q**GNG**EY**T**Y**F**K**S**M-**NS**S**LGL**

MER324279/124-393 TPNG**V**I**V**S**FGY**N---------KGGG--VIW**YDNVTI**-HVN---DVTS**A**Y**MLIDP**MN---**MTG**D-----N-HFY**DLELVFGG**E**SNG**EY**T**Y**F**N**S**L-**NA**S**LAL**

MER324274/148-427 VPNG**V**V**V**S**FGY**Y---------NGT---LYW**YDN**A**TI**-SAP---GVTS**A**Y**MMVSG**FN---**YTG**N-----GYTYY**DAELVFGG**D**ANG**EF**T**Y**F**T**S**M-**NS**T**LSL**

MER093711/139-414 VPQGIE**V**M**FG**FY---------NGT---MNW**YDNVTI**-HES---GVTS**A**Y**MLVDG**FN---**TTG**R-----G-VRY**DAELVFGG**G**GSG**EI**T**N**F**N**S**M-**NS**T**LSL**

MER324275/142-417 SSNA**V**Y**I**S**FGY**S---------LGESEEITW**YDKVII**-PQT---GITS**A**S**FIISG**FS---Q**TP**S-----G-HFY**D**S**ELVFGG**E**GN**REI**T**N**F**T**K**M-**NA**Q**L**M**L**

MER014007/112-404 TSQG**P**M**I**S**FGY**M---------NQSGS-PIW**YDNVTI**-LIP---NTLS**A**Y**ILVDG**YN---**FTA**G-----G-LAY**DAELILGG**G**GNG**EF**T**F**F**N**E**S-**NV**E**LA**M

MER308352/118-388 TSQG**P**M**I**S**FGY**M---------NQSGS-PIW**YDNVTI**-LIP---NTLS**A**Y**ILVDG**YN---**FTA**G-----G-LAY**DAELILGG**G**GNG**EF**T**F**F**N**E**S-**NV**E**LA**M

MER182058/114-405 TSQG**P**M**I**S**FGY**M---------NQSGS-PIW**YDNVTI**-LIP---NTLS**A**Y**ILVDG**YN---**FTA**G-----G-LAY**DAELILGG**G**GNG**EF**T**F**F**N**E**S-**NV**E**LA**M

MER247117/114-407 TSQG**P**I**I**L**FGY**M---------NQSGY-PVW**YDNV**S**I**-LIP---GTLS**A**Y**ILIDG**YN---**LTG**N-----N-HSY**DAELILGG**G**GSG**EF**T**F**F**N**E**S-**NV**E**LA**M

MER015873/97-383 IPQG**V**L**V**H**FGY**L-----------NGSMTVW**YDNVTI**-NIS---GIES**A**Y**LLVDG**YN---**VTG**S-----G-NAY**DTELVFGG**I**S**K**G**EI**T**Y**Y**N**S**L-**SA**M**L**YF

MER093712/24-320 TSNS**V**S**I**W**FGY**EILQ------GSQVTSVQY**YDKVTI**-SQP---GIKS**A**E**ITING**NT---**YTP**D-----G-LYY**DAELVWGG**G**GNG**AP**T**Q**F**T**H**L-**NS**S**LGL**

MER324273/36-334 TSSSLS**I**W**FGY**EILQNGK---PVSSPQVQY**YDEVTI**-KQS---GIVS**A**A**ITVNG**NV---**YTP**D-----G-LYY**DAELVWGG**G**GNG**AP**T**Q**F**V**N**L-**ND**T**LGL**

MER277577/121-378 AKGV**V**L**I**S**FGY**VLIQNG----SLEPVRIVWI**DNVTI**RTSQ---PASS**A**S**IA**A**T**IT----**YSS**S-----GFNAL**DAELVFGG**Y-**NG**SV**V**S**F**K**S**V-**SA**E**LGL**

MER277691/117-380 ANGA**V**H**V**D**FGY**LIAQNGA---QLSQSGATW**FDHVTI**TPSQ---PAVS**V**Q**IVVTT**TF---P**TS**G-----G-NAP**DVELVFGG**I**GNG**EI**T**T**F**N**S**L-R**A**S**LGL**

MER075962/125-409 SNGA**V**H**V**D**FGY**VTAQSG----DYRPAIITW**YDNVTI**RTNP---PAVG**A**G**IV**A**TS**SY---**LNG**R-----G-LPLN**VELVF**C**G**F**CC**SEH**A**T**F**S**E**L-E**A**R**LTL**

MER099912/105-397 INDS**V**V**V**M**FGY**AIVRNGS---TYAPPVIKW**FDNVTL**GIK-----AKD**A**L**IV**T**TP**YE---E**TG**G-----G-YAY**DVELVFGG**GF**NG**EQ**T**T**F**E**S**L-**NA**Q**LA**V

MER015869/23-300 SSPYIY**V**SI**GY**G---------NLSDPTSNT**YDTITI**-NVP---NLQN**A**C**IYVSP**-S---**YTG**V-----G-LPYS**VELVWGG**S**GNG**QS**V**A**F**T**N**M-E**S**D**LA**I

MER247092/144-398 QGKG**V**Y**V**T**FG**FSKIQNGQ---EILPPIFTY**YDKVFI**-SCP---NVIS**A**C**IVI**K**P**NF---**T**K**S**L-------NVFS**A**G**LVFG**SYEQLST**V**S**F**N**N**L-**NS**T**LAL**

MER184686/77-307 SAHN**T**E**V**N**FYY**----------QTYNKTTRR**WDSVLY**KKVN-ISNSHG**S**R**IVIAP**A----**ITP**V-----P-TYS**DLE**F**VFAG**P**GD**RSI**D**Y**F**SFL-**NA**H**LGL**

MER099909/26-283 THGYIN**V**NY**WY**RLNN------TRLDTGWVK**YDDVLI**-----KVNSTR**A**Y**IIIGG**FN-----------PGNLSN**DIE**W**V**T**AG**Y**TA**AAQLY**V**F**K**W-**NA**S**M**I**L**

MER324288/158-372 TGYG**V**V**I**N**FGY**ALLRLGN---ETFSPRVNW**F**A**H**E**LI**PILN-----AT**A**Y**IM**T**SP**AE----**GP**Y-----G-WPM**DTELVIGG**P**GNG**GG**V**K**F**R**E**L-**DA**Y**LSL**

MER106523/120-387 DRGV**V**W**I**N**FT**MVFIRSG----GYIPPTVKK**Y**SVK**VF**PPAP----AQS**A**D**IV**A**TA**RY---**LS**RS-----G-GPLL**VEL**G**IG**EV**G**KYYV**T**T**F**Y**S**L-K**A**E**MAL**

Consensus/80% ....s.l.FhY...................aDplhb...........s.bhlss.....hss...........DhEllbtG.ssG..s.h.p..ss.bsL

29 30 31 32 33

34abc5abc6a7abcd890123456789012345678901234a5678901234567890

* *

MER001319/32-330 **YY**---I---**S**-N----GS**I**TP**V**PS**LYTFG**A**DTAEAA**Y**NV**Y**T**TM-NNGVPIAYNGIENLTI

MER324289/76-250 **YF**---L---**N**-A----TK**L**NI**V**PS**A**L**S**V**G**L**DTAESA**Y**GV**V**V**T------------------

MER093714/51-249 **YF**---L---**Q**-G----NN**L**TI**V**PS**A**L**S**V**G**L**DTAESA**Y**GV**---------------------

MER244602/49-247 **YY**---L---**D**-D----GK**L**AV**V**PS**A**L**S**V**G**L**DTAESA**Y**GI**---------------------

MER247115/50-249 **YY**---L---**Q**-Q----GK**L**VIPSS**AFS**V**G**L**DTAESA**Y**GI**---------------------

MER182036/47-250 **YY**---F---**N**-G----NG**L**SI**V**PN**AYSIG**F**DTAEAA**F**GV**KI-------------------

MER014508/48-251 **YY**---F---**N**-G----NT**L**TI**V**PN**AYSIG**F**DTAESA**Y**GV**K**V**-------------------

MER015865/49-249 **YF**---M---**Q**-S----GK**L**SL**V**PY**AYSIG**F**DTAESV**V**GV**R--------------------

MER069605/49-250 **YF**---K---**D**-G----NS**L**TV**V**PS**A**L**S**T**G**L**DTAETV**I**GI**---------------------

MER242906/105-339 **Y**I---W---**N**-G----TA**Y**VRPPA**AWSLG**LS**T**E**ESA**I**A**AAEPAGFSAVVSRG--------

MER037256/35-313 **YY**---W---**N**-G----NN**Y**QE**I**NN**AYDYG**V**DTAETV**S**NV**T**D**TGVYN-ANGSLFAHITSGS

MER020787/67-360 **EY**---W---**N**-G----HN**Y**QE**I**TN**AFNYG**S**DTAE**IIS**NV**Q**S**SAEYYTSNGTIFENVTAGD

MER020798/40-323 **EY**---W---**N**-G----HN**Y**QV**V**SN**AYNHG**S**DTAEGV**C**NV**S**V**S--YNSASMIPMAHIETGN

MER244710/76-347 A**Y**---W---**N**-G----NN**F**QT**V**IN**AYN**S**G**G**DTGETS**N**NV**R**S**GAYYYVYTGQMIAGLHAGS

MER093713/1-275 **FY**---W---**N**-G----HN**F**QE**V**AN**TYNYG**S**DTAETS**S**NV**K**V**LYYYYPYSGLPTGFITTGP

MER069598/61-341 **LY**---W---**N**-G----HN**F**QE**V**EN**AYNFG**S**DTGESA**E**NV**I**D**SYYYYRYTGLPVAGLTAGS

MER015872/47-337 **EY**---W---**N**-G----HN**F**QT**V**EN**AYNFG**S**DTAETV**Q**NV**V**V**TYTDMPFNGTLVSHITTGT

MER247126/53-333 **YY**---W---**N**-G----HN**F**QE**I**KN**AYNFG**S**DTGETS**N**NV**I**A**SGYYIPSSGELTSGLKAGQ

MER277667/42-293 **QY**---L---**S**-S----GR**L**VS**V**PH**AYDYG**A**DTAETV**YKTS**D**-------------------

MER243100/6-269 **QY**---L---**N**-G----TK**L**VS**V**PH**AFDYG**A**DTAETV**T**G**MA**D**TTSGPYTA-----------

MER324286/35-305 S**Y**---W---**N**-G----NN**F**QN**V**ED**AYNFG**C**DT**E**EG**IN**N**AV**V**TPN----------------

MER324287/43-322 D**Y**---W---**N**-G----NN**F**QA**F**QD**AYNYG**C**DT**E**EG**IN**N**AV**V**NPKFGGNGLPFA-------

MER037300/1-287 ------------------------------------------------------------

MER020805/78-357 **RY**MGS-----------SG**Y**VN**V**PS**AYD**V**G**SE**TGETS**E**GV**A**V**SWDNDTAYL----------

MER013858/88-366 **YY**LNST---**T**------GT**Y**QT**V**PS**AFDI**AA**DTGETS**E**GV**A**V**SWSHGPDGY----------

MER013857/71-370 **KY**IPAA-----N(33)QS**Y**VN**V**PS**AFDFG**T**DTGETS**V-----------------------

MER014466/86-385 **KY**IPAG-----Q(19)MD**Y**VN**V**PS**AFDFG**T**DTGETS**I**GV**A**V**AWNSNDQAIL---------

MER013859/74-368 **Y**LYNST---**S**------KA**F**NT**V**RS**AYD**A**G**V**DTGETS**Y**GV**D**V**GWYGNMADLQSGPSLIYGL

MER014464/71-365 **Y**LFNET---A------HA**Y**EP**V**KS**AYDYG**F**DTGETS**Y**GV**N**V**GWSNHTAKLTGGPSLLYGM

MER324284/152-397 **YY**RGPS----------GD**L**IP**M**PS**AWSMG**S**DTGETV**V**N**----------------------

MER324283/145-398 **YY**QGLN----------GD**L**LP**V**PS**AWSFG**S**DTGET**II**N**----------------------

MER099870/119-365 **LH**-------**R**VN----ES**Y**YP**A**PY**TWGIG**TL**TGEAV**A**N**AS**A**LVSNGV-------------

MER324282/174-453 **YF**---K---Y-G----DY**L**VP**I**PY**MWNFG**MH**TAES**IL**N**AS**S**ISIGPGMAFVKNGNEEPT-

MER324281/175-455 **YF**---A(4)Y-D----YY**L**SP**I**PS**MWSFG**S**DTAE**IIY**N**AH**V**IPIGYGSVNIVNGFEN-L-

MER324285/177-456 **YF**---T(4)**S**-D----YY**L**SP**V**PS**MWDFG**T**DTAE**IIY**D**AH**V**SPLNYGSVYVIQGPEK-L-

MER243354/173-432 **YF**---K---**R**-G----DY**L**VP**V**PF**VWSFG**VH**TAEAA**L**G**AN**A**TAVGPGEVYVSAGLE----

MER283589/126-394 **EY**---W---**N**-G----TH**F**AA**V**PS**F**V**NFG**QA**TAETA**S**NI**K**V**VALYGRGANLTRGVYLPEY

MER247134/247-532 **YY**---F---**N**-G----SE**Y**VT**F**PS**YYTFG**A**DTAEAT**S**N**AH**V**TLEQNGMIKISTGSPDYTY

MER247101/203-469 **FY**---L---**K**-K----NT**Y**VT**F**PN**YFTFG**E**DTAE**K**A**V**NI**---------------------

MER093715/210-491 **FY**---Y---**N**------GS**L**HP**F**PS**VYSFG**A**DTAEGT**S**DL**H**V**SL-MNGLVSVSKGQDNPVF

MER324278/228-482 **FY**---Y---**N**------GS**L**RP**F**PS**VYSFG**G**DTAEAS**T**NV**---------------------

MER324280/303-587 **LY**LNRS---**T**------NT**F**MR**F**PS**YYTFG**S**DTAEGA**Y**DL**---------------------

MER228536/300-582 **MY**LDDQ---**T**------NS**F**VN**F**PS**YYAFG**S**DTAEGA**Y**NL**N**V**T------------------

MER184685/337-630 **LY**--KR---**N**-G----T-**L**TM**F**PT**YYTFG**R**DT**E**EGV**Y**NL**Y**T**AV-KNGTGYVNIGNLNP--

MER015845/113-385 **YY**---N---**S**-T----YG**W**TP**F**RS**IYTYG**VN**TGEGV**T**NL**H**V**SL-IHGYANVYVGNETLSL

MER014507/10-308 **LY**---M---**K**-S----GE**W**VP**F**SQ**VYNYG**S**DTAEST**N**NL**Q**V**LIGKNGDAYVTIGRQNPGL

MER182060/17-315 **LY**---M---**K**-N----GK**W**VP**F**SQ**VYNYG**S**DTAEST**N**NL**R**V**TIAKNGDAYVTIGKQNPGL

MER014517/26-324 **LY**---M---**K**-N----GK**W**VP**F**SQ**VYNYG**S**DTAEST**N**NL**R**V**TIAKNGDAYVTIGKQNPGL

MER324276/33-291 **LY**---M---**K**-N----GK**W**VP**F**SQ**VYNYG**S**DTAEST**N**NL**R**V**TIAKNGDAYVTI-------

MER069582/14-314 **FY**---L---**S**-G----NS**F**VP**F**ST**VYTYG**N**DTAEST**G**DL**Y**V**SIASNGDAYVTVGTPDYGL

MER069572/8-307 **YY**---L---**S**-D----QQ**W**KP**F**KN**IFDYG**F**DTAEGA**Y**NL**N**V**SLSNQGYALVRVGSENFQL

MER069601/12-309 **YY**---L---**R**-N----GQ**W**LS**F**NE**IYNYG**F**DTAESA**N**NL**T**V**YVDKQGFAHVITGNLYKGE

MER182056/124-400 **Q**I-ILL---**N**-G----T-NVT**L**SN**LYTFG**S**DT**E**ESA**D**NL**M**T**IY-ENGYAWVVVGND----

MER184687/128-406 **Q**I-ILQ---**N**-G----T-**I**ISPNS**LYTFG**S**DT**E**ESA**D**NL**M**V**LF-QNNYAWVLIGNN----

MER324279/124-393 AM-ELP---**N**-G----N-**Y**VYPYP**LYTFG**S**DT**E**ESA**D**NL**Q**T**VF-VNG-------------

MER324274/148-427 S**Y**-HLL---**N**-G----S-**W**TVPPE**LYGFG**A**DT**E**EAA**D**NL**A**T**VL-VNGVPTVEIGRE----

MER093711/139-414 **MY**--VV---**N**-G----V-NETPQE**LYGYG**A**DTAEAA**D**NL**K**T**TL-VNGVPTVTLGQE----

MER324275/142-417 **KY**-VLE---**N**-E----T-**I**VLPMS**LYGFG**S**DTAEGA**D**DL**S**T**NL-IDGIP-----------

MER014007/112-404 **IY**-QYL---**N**-G----T-**L**APPKF**LFPFG**L**DT**E**ESA**D**NL**Y**S**IS-YNGVYLVSSGYQVINN

MER308352/118-388 **IY**-QYL---**N**-G----T-**L**APPKF**LFPFG**L**DT**E**ESA**D**NL**Y**S**IS-YNGVYLVSSG------

MER182058/114-405 **IY**-QYL---**N**-G----T-**L**APPKF**LFPFG**L**DT**E**ESA**D**NL**Y**T**VS-YNGVYLATNGYQVINN

MER247117/114-407 **IY**-QYL---**N**-G----T-**L**APPKY**LFPFG**L**DT**E**ESA**D**NL**Y**T**IA-YKGAYLVSTGYQVINN

MER015873/97-383 V**Y**---V---**N**-S----TNVITPKA**L**L**PFG**L**DTAEAS**D**NL**F**T**TP-YQGVYKVKVGNGEETY

MER093712/24-320 **YY**---L---**S**-S----TGVTP**I**PS**LYTFG**SN**TGESA**Y**NV**H**D**NL-VNGVPESYVGTEELTI

MER324273/36-334 **YY**---V---**N**ST----GK**I**TP**F**PS**LYTFG**S**DTGESA**Y**NV**H**D**ML-VNGVPEAYAGTEVLTV

MER277577/121-378 **FY**---W---**D**-G----AG**W**TA**F**PN**LYNFG**VNV**PEAA**S**NL**A**V**QY-YGGLAHVGVGALSPSS

MER277691/117-380 **YY**---L---**N**-G----SS**W**VP**L**PY**LYTYG**A**DTAEGA**T**DL**A**T**SW-GNGLASVAVGPLSPGR

MER075962/125-409 A**Y**---W---**R**-G----GG**W**AP**F**PN**LYSFG**VS**TNETA**T**NV**A**V**RY-ADGFAVVERGALSPAK

MER099912/105-397 **MH**---W---**S**-G----SG**W**VP**Y**SQ**VYNFG**MN**TGESA**T**DL**V**T**SISSNGNVQVTVGIPYYGE

MER015869/23-300 **FY**--QV---**K**-P----EL**Y**SP**F**PI**IYNYG**F**DT**Q**ESA**S**DL**S**A**YLASDGLVEVSVGTPNPTL

MER247092/144-398 **LY**---C---**N**------QG**W**KP**V**PS**FFSF**CVNSL**ESA**C**NL**NFKAAKS--------------

MER184686/77-307 **FY**FNNT---**T**------NS**Y**AD**F**PE**YFTKG**R**DTGEVS**Y**DL**K**S**TISNDQATVQTDMP-----

MER099909/26-283 **MY**-------**R**YN----GS**W**FTPPG**G**Q**SR**SF**DTGESV**N**PI**A**G**ISESYING-----------

MER324288/158-372 **LY**---W---**N**-G----TS**W**AP**Y**PI**TYTFG**VS**TGE**Y**A**V**NV**Y**V**-------------------

MER106523/120-387 V**F**---W---**D**-G----SA**W**RP**F**PD**LYSFG**VN**TG**GW**P**T**NV**V**V**SYRGGYAYLNASAGLKPQR

Consensus/80% ba.......p........b..h..hasbG.DTsEss.sl.s...................
